# Functionally Deregulated AML1/RUNX1 Cooperates with BCR-ABL to Induce a Blastic Phase-Like Phenotype of Chronic Myelogenous Leukemia in Mice

**DOI:** 10.1371/journal.pone.0074864

**Published:** 2013-09-30

**Authors:** Kiyoko Yamamoto, Shinobu Tsuzuki, Yosuke Minami, Yukiya Yamamoto, Akihiro Abe, Koichi Ohshima, Masao Seto, Tomoki Naoe

**Affiliations:** 1 Division of Molecular Medicine, Aichi Cancer Center Research Institute, Nagoya, Japan; 2 Department of Hematology and Oncology, Nagoya University Graduate School of Medicine, Nagoya, Japan; 3 Department of Hematology, School of Medicine, Fujita Health University, Toyoake, Japan; 4 Department of Pathology, School of Medicine, Kurume University, Kurume, Japan; B.C. Cancer Agency, Canada

## Abstract

Patients in the chronic phase (CP) of chronic myelogenous leukemia (CML) have been treated successfully following the advent of ABL kinase inhibitors, but once they progress to the blast crisis (BC) phase the prognosis becomes dismal. Although mechanisms underlying the progression are largely unknown, recent studies revealed the presence of alterations of key molecules for hematopoiesis, such as *AML1/RUNX1*. Our analysis of 13 BC cases revealed that three cases had *AML1* mutations and the transcript levels of wild-type (wt.) *AML1* were elevated in BC compared with CP. Functional analysis of representative AML1 mutants using mouse hematopoietic cells revealed the possible contribution of some, but not all, mutants for the BC-phenotype. Specifically, K83Q and R139G, but neither R80C nor D171N mutants, conferred upon BCR-ABL-expressing cells a growth advantage over BCR-ABL-alone control cells in cytokine-free culture, and the cells thus grown killed mice upon intravenous transfer. Unexpectedly, wt.AML1 behaved similarly to K83Q and R139G mutants. In a bone marrow transplantation assay, K83Q and wt.AML1s induced the emergence of blast-like cells. The overall findings suggest the roles of altered functions of AML1 imposed by some, but not all, mutants, and the elevated expression of wt.*AML1* for the disease progression of CML.

## Introduction

BCR-ABL generated by the chromosomal translocation t(9;22)(q34;q11) in hematopoietic stem cells constitutively activates tyrosine kinase on its own and leads to CML [1]. Notwithstanding the remarkable success in treating patients in CML-CP with ABL kinase inhibitors such as imatinib [2], [3], some patients acquire resistance or intolerance to ABL kinase inhibitors, culminating in disease progression from CML-CP to the accelerated phase (AP) and BC [1], [3], [4]. Mechanisms responsible for the disease progression remain largely unknown, but likely involve activation of oncogenes, inactivation of tumor suppressors, and impairment of differentiation [3].

Although BCR-ABL plays a central role in the pathogenesis of CML-CP, the unrestrained expression and continuous activity of BCR-ABL kinase itself are thought to accelerate the disease [3]. Specifically, BCR-ABL-induced endogenous reactive oxygen species cause chronic oxidative DNA damage that result in double-strand breaks (DSBs) in S and G2/M cell-cycle phases [3], [5]. Although homologous recombination and nonhomologous end-joining represent 2 major mechanisms of DSB repair, these repair mechanisms are not perfect in BCR-ABL positive cells [5] and lead to a variety of point mutations and chromosomal aberrations [3], [5].

*AML1*, a founding member of the *RUNX* family [6]–[8], is required for the emergence of definitive hematopoiesis [9] and regulates transcription of genes important for hematopoiesis [7], [8]. Functional deregulation of AML1 by chromosomal translocations and somatic point mutations is commonly involved in hematological malignancies. In fact, *AML1* is the most frequent target gene of chromosomal translocation associated with human leukemia [7], [8], [10], and *AML1* point mutations have been often identified in acute myeloid leukemia (AML), myelodysplastic syndrome (MDS), and CML-BC [11]–[19]. These altered AML1s are supposed to dominant-negatively inhibit the function of wild-type (wt.) AML1, thereby blocking myeloid differentiation [7], [12]–[15]. Moreover, targeted *AML1* deletion in established hematopoietic stem cells leads to an expansion of hematopoietic progenitor cells. These findings imply that impaired AML1 functions may enhance self-renewal of progenitor cells and block their differentiation, thus priming the cells for leukemic transformation [7], [8], [20]. One might expect that such altered functions of AML1, coupled with the accelerated cell growth by BCR-ABL, induce CML-BC. AML1-EVI1 [21] or AML1-MDS1-EVI1 fusion [22] exemplifies such a synergism. *AML1-MDS1-EVI1* inhibits differentiation of 32Dcl3 [23], [24] and mouse bone marrow cells. Although an extended latency is required for *AML1-MDS1-EVI1* to elicit leukemia [25], coexpression of BCR-ABL has facilitated the development of AML-like disease in mice [26].

Recently, mutations of genes crucial for hematopoiesis (*RUNX1, ASXL1, WT1, NRAS, KRAS, TET2, CBL, TP53, IDH* and *IKZF*) were reported to occur in 76.9% of CML-BC patients. Significantly, *AML1* mutations account for 33.3% of CML-BC patients, while no such mutation was detected in CML-CP samples [16], suggesting that *AML1* mutants contribute to the transition from CML-CP to CML-BC. Although somewhat paradoxically, experimental expression of wt., but not mutant, *AML1* protects BCR-ABL-transformed cells from imatinib-induced apoptosis, indicating the role of up-regulated wt.AML1 in imatinib resistance and disease progression of CML [27].

We hypothesized that the deregulated activity of *AML1*, either a loss or gain of function, may represent one of the mechanisms for disease progression of CML, and set out to investigate expression levels of AML1 and its mutation in CML-BC for comparison with CML-CP clinical samples. We then explored the possible contribution of AML1s (wt. and mutants) to the BC phenotype using mouse cells *in vitro* and *in vivo*.

## Materials and Methods

### Mice

C57BL/6N mice were purchased from Charles River (Atsugi, Japan) and SLC (Hamamatsu, Japan). NOD/SCID and NSG (NOD. Cg-*Prkdc^SCID^* Il2*rg*t^m1Wjl^/SzJ) mice were purchased from Charles River and Jackson Laboratory (Sacramento, CA), respectively. All animal experiments were performed according to protocols (Permit Number: 22-7) approved by the Institutional Animal Care and Use Committee at the Aichi Cancer Center.

### Patient Samples and Cell Lines

CML-CP and CML-BC samples were obtained with the approval of the Institutional Review Boards at Aichi Cancer Center and Nagoya University. Written informed consent was obtained in accordance with the Declaration of Helsinki. Normal peripheral blood (PB) and bone marrow (BM) cells were obtained from healthy volunteers. CML cell lines used were as follows: K562 (ATCC-CLL-243), Nalm1 (ATCC-CRL-1567), BV-173(DSMZ ACC-20), MegO1 [28] (provided by Dr. Michinori Ogura, Nagoya University), and MegA2 [29] (provided by Dr. Akihiro Abe, Fujita Health University).

### Plasmids

MSCV-p210*BCR/ABL*-IRES-GFP plasmid [30], cDNAs for *AML1b* [31], *AML1R139G* [14], *NUP98-HOXA9* [32], *Bmi1* [33] and *Hes1* [34] were kindly provided by Dr. Warren Pear (University of Pennsylvania, Philadelphia, PA), Dr. Misao Ohki (National Cancer Center, Tokyo, Japan), Dr. Hisamaru Hirai (University of Tokyo), Dr. Nabeel R. Yaseen (Washington University School of Medicine, St. Louis, MO), Dr. Toshinori Nakayama (Chiba university, Chiba, Japan) and Dr. Toshio Kitamura (University of Tokyo) respectively. AML1 mutants were generated using a mutagenic PCR method. MSCV-puro^R^ (Clontech) and MSCV-IRES-human(h)CD8 [35] were used to express wt. or mutant AML1 along with puromycin-resistant gene or an extracellular domain of human CD8. CShU6PIG shRNA vector has been described [35].

### Flow Cytometric Analysis, Progenitor Cell Isolation and Retroviral Infection

Anti-Gr-1 (RB6-8C5), anti-Mac-1(M1/70), anti-B220 (RA3-6B2), anti-CD3 (145-2C11), anti-TER-119 (TER-119), anti-CD41 (eBioMWReg30), anti-c-kit (2B8), anti-Sca-1 (D7) (eBioscience, San Diego, CA) and anti-Fc receptor (2.4.G2; BD Biosciences, Franklin Lakes, NJ) antibodies were used. Flow cytometry was performed on FACSCalibar (BD Bioscience) and JSAN (BayBioscience, Kobe, Japan) devices. Data were analyzed using Flow Jo software ver.7.6.2 (Tree Star, Ashland, OR). Lineage marker-negative (Lin-)/Sca-1-negative (Sca-1-) e14 C57BL/6N mouse fetal liver cells were isolated using antibodies for Gr-1/Mac-1/CD3/B220/Ter-119/Sca-1 and MACS columns (Miltenyi Biotec, Bergisch Gladbach, Germany). Cells were spin-infected with retrovirus, as described [36].

### In vitro Liquid Culture and Colony-forming Assay

Cells (1×10^5^) were grown in Iscove’s Modified Dulbecco Medium (IMDM) supplemented with 10% fetal calf serum (FCS) without cytokines, in triplicate, and counted using the trypan blue exclusion method. For the colony-forming assay, 1×10^4^ cells were plated in triplicate, as described [36], without cytokines, and replated every 7 days. Images of colonies were obtained using a CKX31 microscope (OLYMPUS) with a SPlan objective lens (OLYMPUS) and a C-4040 camera (OLYMPUS), and processed using Photoshop software ver.6 (Adobe, San Jose, CA).

### sh RNA for Knocking down *AML1*

Target sequences for shRNA were 5′-CCTCGAAGACATCGGCAGAAA-3′ [37] and, 5′-ACTTTCCAGTCGACTCTCA-3′ [38] for *AML1*, and 5′-CTTACGCTGAGTACTTCGA-3′ for luciferase (control).

### Quantitative RT-PCR

Quantitative PCR was performed as described [39]. Primers used were 5′-TGTCGGTCGAAGTGGAAGAGGGAA-3′/5′-AGCTCCCGGGCTTGGTCTGA-3′ for human *AML1* and 5′-GCGGGAAATCGTGCGTGACATT-3′/5′-GATGGAGTTGAAGGTAGTTTCGTG-3′ for human *β-actin.*

### Western Blot Analysis

Anti-AML1 (D33G6: Cell Signaling Technology, Danvers. MA) and anti-tubulin (T9026: Sigma, St. Louis, MO) antibodies were used.

### Mutation Analysis of the *AML1* Gene

cDNAs for *AML1* were amplified using RT-PCR and subjected to direct sequencing as described [15].

### DNA Binding Ability Assay

A nuclear extract of 293T cells transfected with Myc-tagged CBFβ was mixed with that of cells transfected with either FLAG-tagged wt. or mutant AML1s, and then incubated with annealed 5′-biotinylated oligo-DNAs for the binding site of AML1. The DNA was captured by streptavidin-agarose beads, and co-purified proteins were analyzed by immunoblotting with anti-FLAG antibody, as described [39].

### Transcriptional Assay

293T cells were transfected with plasmids for the luciferase reporter containing macrophage colony-stimulating factor receptor (*M-CSFR*) promoter (M-CSF-R-luc) [12], CBFβ, RL-Tk (Promega), and FLAG-tagged wt. and mutant AML1s. Luciferase reporter activities were measured using a Dual Luciferase Assay System (Promega), and normalized on the basis of the activities of pRL-Tk (internal control).

### Transplantation Assay

Cultured cells were intravenously transferred into lethally irradiated syngeneic mice, along with 0.5×10^6^ of radioprotective fresh BM cells. In some experiments, BM cells were isolated from C57BL/6N mice that received 5-fluorouracil (150 mg/kg) 4 days earlier, and then cultured in the presence of SCF (stem cell factor), IL-3, IL-6 and 1×10^−5^ M hydrocortisone, and infected with retrovirus, as described in the literature [34]. Cells co-transduced with MSCV-p210*BCR-ABL*-IRES-GFP and either MSCV-wt.*AML1*-IRES-hCD8, MSCV-*AML1K83Q*-IRES-hCD8 or MSCV-*AML1R80C*-IRES-hCD8 were transplanted intravenously into 2.5 Gy-irradiated NOD-SCID or NSG mice. Probabilities of event-free survival were estimated using the Kaplan-Meier method, and statistical analysis was performed using the log-rank test with *post hoc* analysis using Holm’s method.

## Results

### *AML1* Expression is Elevated in CML-BC Patient samples and Knocking down of AML1 Inhibits Growth of Human CML Cell Lines

**Figure 1:**
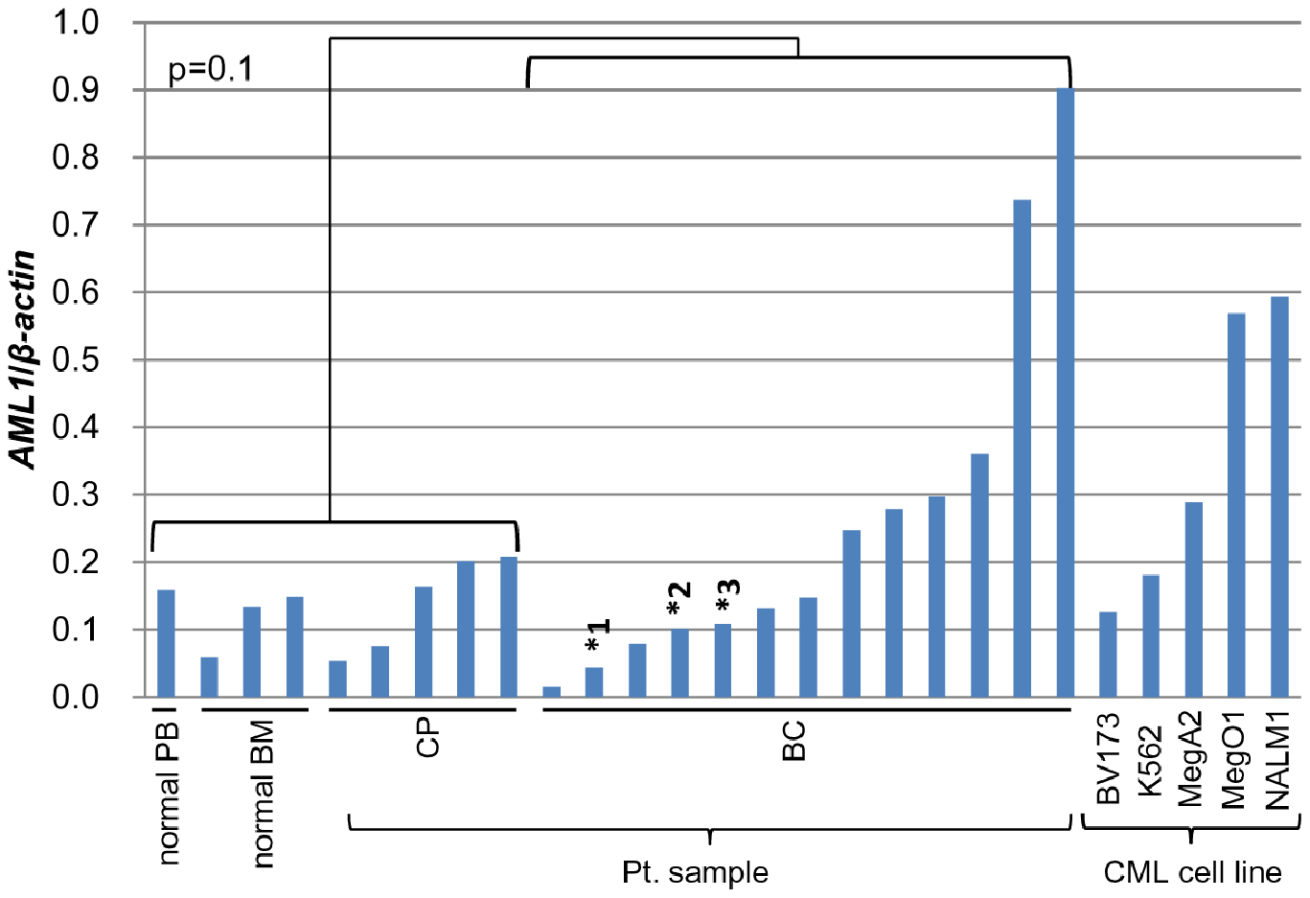
*AML1* transcript levels in CML-BC patient samples. Quantitative RT-PCR of *AML1* mRNA levels of normal subjects, CML patient samples, and CML cell lines. Transcript levels of *AML1* normalized on the basis of those of *β-actin* are presented. *1, *2 and *3 indicate cases with *AML1* mutation *R135EfsX42*, *A297LfsX7* and *G190R*, respectively.

**Figure 2:**
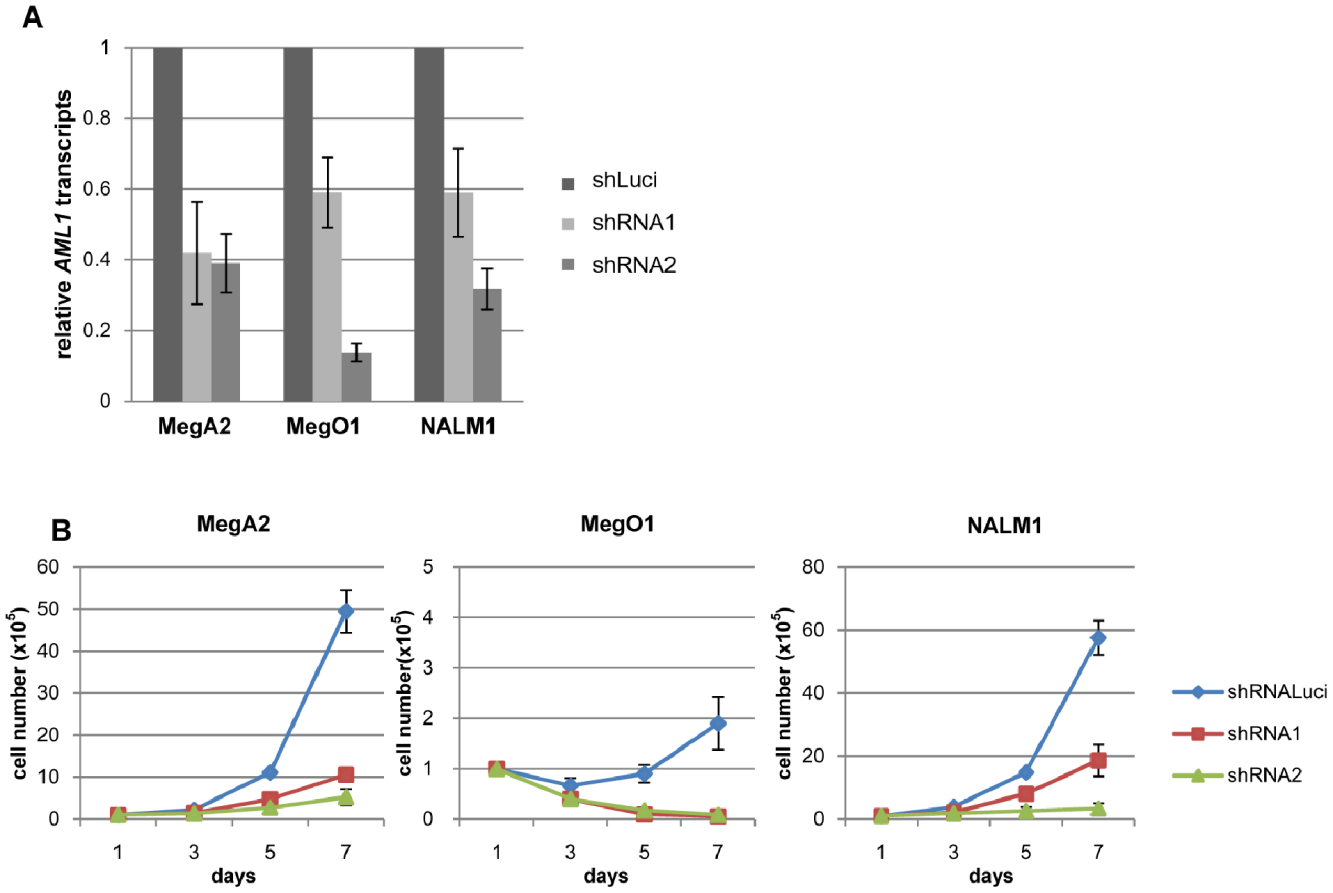
Effects of knocking down AML1 in human CML cell line. (A) Quantitative RT-PCR of *AML1* transcripts in CML cell lines infected with shRNA viruses for luciferase (control) and *AML1* (shRNA-AML1–1 and shRNA-AML1–2). Two different viruses were used for *AML1* silencing and effectively silenced AML1 expression. Results are normalized on the basis of *β-actin* transcript levels, and are presented as relative values to those of the control. (B) Growth curves of MegA2, MegO1 and Nalm1 cell lines infected with shRNA viruses for luciferase and *AML1*. Viable cells were counted every other day with the use of trypan blue.

We first examined transcript levels of *AML1* and its mutations, in clinical samples of 13 CML-BC (Table S1) and 5 CML-CP cases, 5 CML-BC cell lines (K562, MegO1, MegA2, Nalm1 and BV173), and 4 normal blood samples (3 BM and one PB). Although there was no statistically significant difference in *AML1* levels between CML-BC and CML-CP or normal blood samples, noteworthy are the four outlier BC patients whose *AML1* levels exceeded the mean +2SD of those of CML-CP samples (Figure 1). Investigation using data of a large number of cases made available by others [40] revealed that *AML1* transcript levels were higher in CML-BC than CML-CP patients and the difference was statistically significant (p<0.001) (Figure S1). Direct sequencing of coding regions of *AML1* amplified by RT-PCR revealed the presence of mutations in 3 (25%) of 12 evaluable CML-BC patients: *G190R*, *R135EfsX42* and *A297LfsX7*. *AML1* mutations were not found in any of the 5 CML-BC cell lines examined (exons 3–5 of MegA2 were excluded because of the consistent failure of RT-PCR-mediated amplification). Interestingly, the four outlier CML-BC cases had no mutation in the *AML1* gene, raising the possibility that some CML-BC might be causally related to elevated expression of wt.AML1. To gain insights into this possibility, we chose three cell lines MegA2, MegO1 (megakaryocytic) and Nalm1 (B lymphoid) that exhibited higher *AML1* transcript levels compared with those of normal and CML-CP samples, and examined the effects of shRNA-mediated silencing of AML1 (Figure 2). Two shRNAs viruses for AML1 effectively knocked down AML1 expression (Figure 2A, Figure S2) and inhibited the growth of cells (Figure 2B). Cell death was also observed in MegO1 (data not shown). Importantly, *AML1* transcript levels obtained following infection with shRNA virus in these cell lines were comparable to those of CML-CP patients (Figure 1 and Figure 2A). These finding suggest that over-expression of AML1 plays important roles in the growth of CML-BC cells.

### DNA Binding and Transcriptional Activity of Mutant AML1s

**Figure 3:**
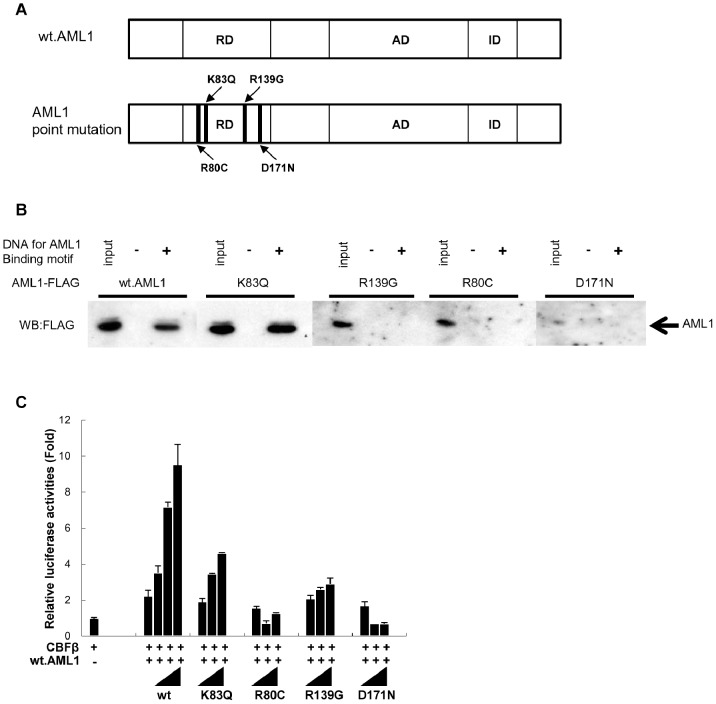
DNA binding and transcriptional activity of *AML1* mutants. (A) Schematic diagram of wt.AML1 and AML1 mutants. RD: runt domain, AD: transcription activation domain, ID: transcription inhibition domain. (B) DNA binding activity of AML1 mutants. Nuclear extracts of cells programmed to express CBFβ and the indicated AML1 were incubated with biotinylated double-stranded oligo-DNA for the AML1 binding motif. The DNA-bound AML1 mutants or wt.AML1 protein were captured by streptavidin agarose and visualized by immunoblotting with anti-FLAG antibody. (C) Transactivation assay of AML1 mutants. 293T cells were transfected with M-CSF-R-luc reporter plasmid (0.5 µg), expression plasmid for CBFβ (0.5 µg), plasmid for wt.AML1 (3 µg), graded amounts of plasmids for wt.AML1 (3, 6, 9 µg) or AML1 mutants (3, 6, 9 µg), and Renilla luciferase plasmid (0.01 µg). Luciferase activities are normalized on the basis of Renilla luciferase activity. Results are presented as a fold change relative to the activity observed by CBFβ alone (average±SD.). Representative results from 3 independent experiments are shown.

We next investigated the DNA-binding and transactivation abilities of BC-related AML1 mutants. For this purpose, we chose AML1K83Q and AML1R80C mutants (Figure 3A) because they have been reported to occur recurrently in BC (Table S2). We also included AML1R139G and AML1D171N in our analysis (Figure 3A); mutations involving R139 are known in CML-BC [17], [18] and AML/MDS [14], and AML1D171N as found in an AML/MDS patient [15], [19] induces MDS/AML in a mouse BM transplantation (BMT) model [13]. Nuclear extracts of 293T cells programmed to express CBFβ and AML1 mutants were incubated with biotinylated double-stranded DNA for the AML1 binding motif. The DNA was then captured using streptavidin agarose beads, and protein co-purified was detected by Western blot analysis. Results showed that while the DNA binding ability of AML1K83Q was comparable to that of wt.AML1, the remaining 3 mutants (AML1 R80C, R139G, and D171N) did not show appreciable DNA binding ability (Figure 3B), which is consistent with reported findings [12], [14], [19]. These mutants were then tested for their transcription abilities using a reporter construct based on M-CSF-R [12], which is a well-characterized myeloid-specific AML1 target. Competition experiments in which mutant and wt.AML1 constructs were cotransfected in varying ratios (Figure 3C) revealed that AML1K83Q has the capacity to enhance the reporter activity, albeit to a lesser degree compared with wt.AML1. AML1R139G slightly, but significantly, enhanced the reporter activity at high doses (Figure 3C). In contrast, AML1R80C and AML1D171N suppressed the transactivation activity of wt.AML1 (Figure 3C). In the following studies, we compared these AML1 mutants and wt.AML1 harboring different DNA-binding and transcriptional properties with respect to co-operability with BCR-ABL.

### AML1 Mutants and wt.AML1 Cooperate with BCR-ABL to Immortalize Hematopoietic Progenitor Cells *in vitro*

To determine whether AML1 mutants play a role in disease progression of CML, we adopted and modified a culture system to assay the ability of a given gene to collaborate with BCR-ABL to induce cell proliferation [34], [41]. We first chose *NUP98-HOXA9* [41], [42], *Bmi1* [43], [44] and *Hes1* [34] (all known to induce BC in mice) to transduce Lin-/Sca-1- progenitor cells derived from mouse fetal liver together with *BCR-ABL*. Cells were then cultured in a cytokine-free condition. *BCR-ABL-*alone-transduced cells grew initially, but ceased to do so after a week (Figure S3), as observed in previous reports [34], [45]. In contrast, cells co-transduced with *BCR-ABL* and either *NUP98-HOXA9, Bmi1* or *Hes1* continued to grow for at least 11 days (Figure S3), demonstrating the utility of the assay system to facilitate identification of genes that may contribute to the development of BC.

**Figure 4:**
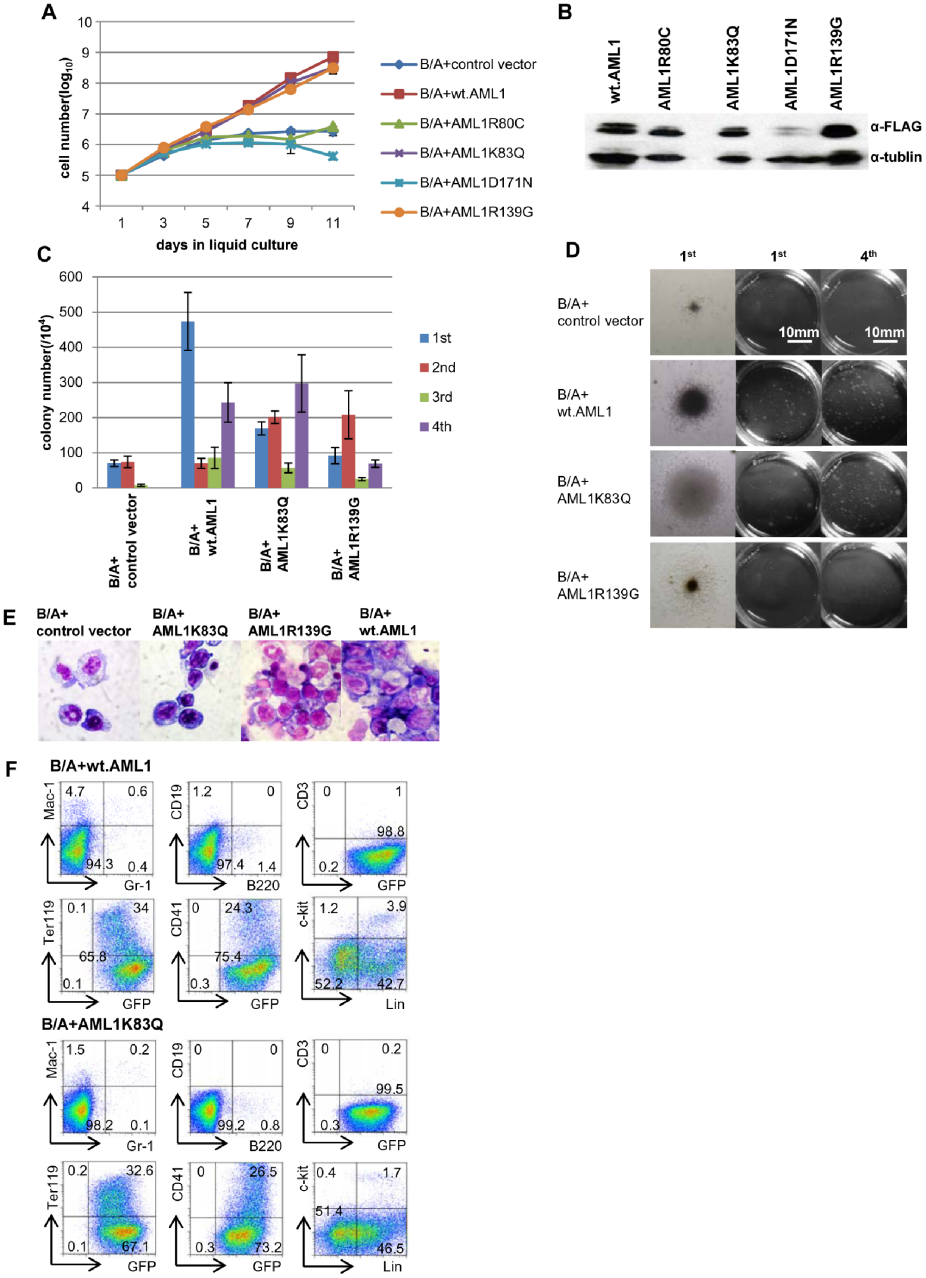
Cytokine-independent growth of progenitor cells co-transduced with *BCR-ABL* and wt.*AML1* or *AML1* mutants. (A) Growth curves of progenitor cells co-transduced with *BCR-ABL* (B/A) and the indicated *AML1s* in liquid culture. Cells were counted every other day with the use of trypan blue and data are presented as average±SD. Representative results from 2 independent experiments are shown. (B) Western blot analysis of Lin-, Sca-1- progenitor cells transduced with *BCR-ABL/AML1* mutants and *BCR-ABL*/wt.*AML1* using anti-FLAG and anti-tubulin antibody. (C, D) Colony-forming abilities of Lin-, Sca-1- progenitor cells co-transduced with *BCR-ABL* and either the indicated *AML1* or vector-only in the absence of cytokines. Colony numbers are presented as average±SD. Two independent experiments yielded a similar result (C). Photomicrographs of colonies of cells co-transduced with *BCR-ABL* and either the indicated *AML1* or vector-only in the 1^st^ and 4^th^ rounds of replatings. Original magnification: ×100 (left). A scale bar is presented (middle and right) (D). (E) May-Grünwald-Giemsa staining of Lin-, Sca-1- progenitor cells transduced with *BCR-ABL/AML1K83Q*, *BCR-ABL/AML1R139G*, *BCR-ABL/wt.AML1*, and *BCR-ABL*/control vector. Original magnification: ×1000. (F) Flow cytometric analysis of Lin-, Sca-1- progenitor cells grown in liquid culture that were transduced with *BCR-ABL/AML1K83Q* or *BCR-ABL/wt.AML1* and cultured in the absence of cytokines.

We then tested wt.AML1 and AML1 mutants for their co-operability with BCR-ABL. Cells co-transduced with *BCR-ABL* (that co-expresses GFP) and either mutant or wt.*AML1* (that co-expresses the puromycin-resistant gene) or vector-only control were selected for puromycin-resistance, and cultured without cytokines. GFP-positive cells in this culture therefore represent BCR-ABL- and mutant/wt.AML1-doubly transduced cells, while GFP-negative cells represent mutant/wt.AML1-alone-transduced cells. Results showed that while *BCR-ABL/AML1K83Q* and *BCR-ABL*/*AML1R139G* cells continued to grow for at least 11 days, *BCR-ABL*/*AML1R80C* and *BCR-ABL*/*AML1D171N* cells lacked such an ability (Figure 4A). *BCR-ABL/*wt.*AML1* cells also grew continuously (Figure 4A). Western blot analysis confirmed expression of AML1s in the cells (Figure 4B), with the expression levels of AML1R80C, AML1K83Q, and AML1R139G being similar, although the expression level of AML1D171N was somewhat lower than that of the other mutants. Cells transduced with wt.AML1 or mutant AML1s alone rapidly underwent cell death (Figure S4). Accordingly, while GFP positivity was 20–30% at the beginning of culture, it was nearly 100% in cells that gained a growth ability after 7 days of cytokine-free culture (data not shown). These results raise the possibility that AML1K83Q, AML1R139G and wt.AML1 cooperate with BCR-ABL to confer a growth-promoting capacity upon progenitor cells in the absence of cytokines.

The findings obtained using liquid culture were additionally confirmed by a colony-forming assay. The replating assay revealed that *BCR-ABL*-only-transduced progenitor cells generated colonies up to the 3^rd^ plating, but virtually yielded no colonies in the 4^th^ plating (Figure 4C, D). In contrast, cells co-transduced with *BCR-ABL* and either *AML1K83Q*, *AML1R139G* or wt.*AML1* efficiently generated colonies even at the 4^th^ plating (Figure 4C, D). In addition, the number of colonies was significantly increased and the sizes of colonies were considerably larger in comparison to *BCR-ABL*-only-transduced control cells (Figure 4D, left). Cytospin preparations of cells that acquired a proliferation capacity in the liquid culture showed that *BCR-ABL*/*AML1K83Q-, BCR-ABL*/*AML1R139G-* and *BCR-ABL*/wt.*AML1*-cotransduced cells were morphologically compatible with blasts that exhibit a high nuclear-cytoplasmic (N/C) ratio (Figure 4E). While the majority of these cells expressed Ter119 and CD41, cells expressing Gr-1, Mac-1 or c-kit were barely detectable and approximately 50% of cells were Lin- immature progenitor cells (Figure 4F). In contrast, *BCR-ABL*-only cells were largely morphologically differentiated cells such as macrophages (Figure 4E). These data indicate that AML1K83Q, AML1R139G and wt.AML1, but not AML1R80C or AML1D171N, cooperate with BCR-ABL to confer a proliferative capacity on progenitor cells in the absence of cytokines. The proliferating cells thus generated were a mixture of Lin- progenitor cells and cells with megakaryocyte/erythroblast-affiliated markers.

### AML1 Mutants and wt.AML1 Cooperate with BCR-ABL to Induce CML-BC-like Disease in Mice

To test the co-operability of AML1K83Q, AML1R139G and wt.AML1 with BCR-ABL *in vivo*, we intravenously transferred cells grown in cytokine-free culture (shown in Figure 4A) into lethally irradiated mice with radio-protective fresh BM cells. Recipients of *BCR-ABL*-only-transduced cells (n = 2) survived without any signs of disease for at least 4 months (Figure S5), at which time the mice were found to have no detectable BCR-ABL+ cells in BM or spleen upon sacrifice, consistent with reported findings [41]. In contrast, all recipients of cells co-transduced with *BCR-ABL* and *AML1K83Q* (n = 5) or wt.*AML1* (n = 5) became moribund or died within 30 days after the transfer. *BCR-ABL*/*AML1R139G*-co-transduced cells were less potent in the lethality (Figure S5), probably reflecting low clonogenicity as assayed *in vitro* (Figure 4C, D). One of 6 recipients survived beyond 4 months of the observation period, and the survival was prolonged compared with that of cases involving of *BCR-ABL/AML1K83Q* or *BCR-ABL*/wt.*AML1* (median event-free survival was 78, 18 and 14 days for *BCR-ABL*/*AML1R139G*, *BCR-ABL*/*AML1K83Q*, and *BCR-ABL*/wt.*AML1*, respectively) (Figure S5). All diseased mice developed Ter119- and/or CD41-positive erythro/megakaryocytic leukemia, except that one mouse transplanted with BCR-ABL/AML1R139G-cotransduced cells developed B lymphoid leukemia.

**Figure 5:**
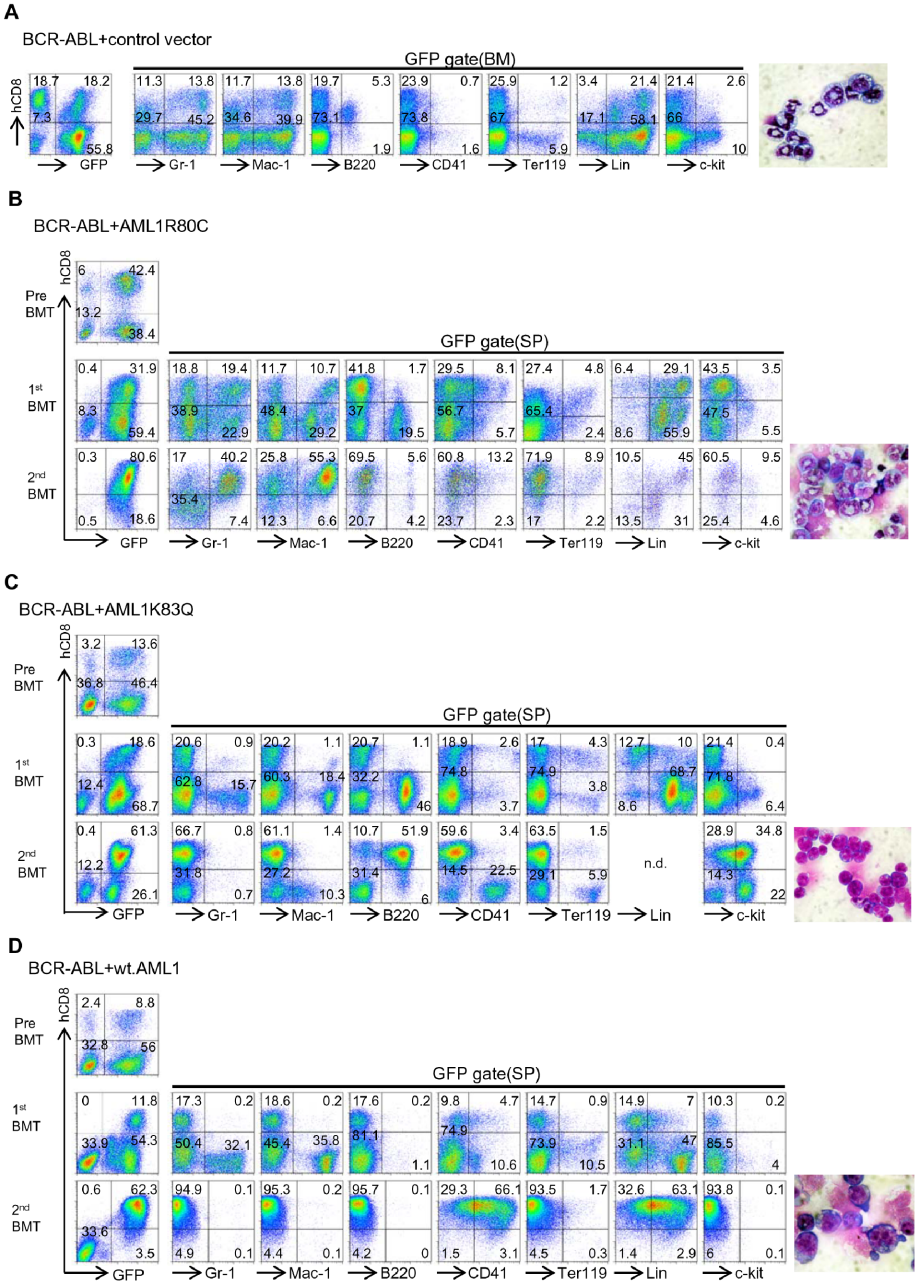
AMLK83Q mutants and wt.AML1 cooperate with BCR-ABL to induce a CML-BC-like phenotype in mice. Flow cytometric analysis of bone marrow (BM) or spleen (SP) cells from mice receiving BM cells transduced with a combination of *BCR-ABL* and either indicated *AML1* mutant or wt.*AML1*. *BCR-ABL*- and *AML1*-transduced cells are marked by GFP and human CD8, respectively, as surrogated markers. GFP+ cells were also analyzed for expression of hCD8 versus Gr-1, Mac-1, B220, CD41, Ter119, a mixture of lineage marker, and c-kit. Photomicrographs of cells are also presented: May- Grünwald-Giemsa staining, original magnification ×1000. (A) Morphologies of BM cells (right) and flow cytometric analysis of BM cells from a primary recipient of *BCR-ABL*/control-transduced cells. (B, C, D) Flow cytometric analysis of cells from primary (1^st^) and secondary (2^nd^) recipients of *BCR-ABL/AML1R80C-* (B), *BCR-ABL/AML1K83Q-* (C), or *BCR-ABL/wt.AML1-* (D) transduced cells. Expression of GFP and hCD8 on cells before transplantation are presented at the top-left of each result, and expression of GFP and hCD8 on SP cells from recipient mice are presented at the left. GFP+ SP cells were analyzed for the expression of hCD8 versus the indicated marker (remaining panels). Morphologies of spleen cells from secondary recipients are also presented (far right).

We then chose *AML1K83Q* and wt.*AML1* to investigate their co-operability with *BCR-ABL* using a conventional BMT assay. To this end, retroviruses for wt. and mutant *AML1*s that co-express an extracellular domain of human CD8 (hCD8) by virtue of an IRES element were used to facilitate identification of infected cells. *AML1R80C* was also included in the analysis, because this mutant showed, unlike *AML1K83Q*, dominant-negative activity against wt.*AML1* in the transactivation assay conducted in Figure 3C, and exhibited a different activity compared with *AML1K83Q* and wt.*AML1* in culture (Figure 4A). BM cells harvested from 5-FU-treated C57BL6N mice were cotransduced with *BCR-ABL* and either *AML1K83Q,* wt.*AML1, AML1R80C,* or hCD8-only control, and intravenously transplanted into sublethally irradiated mice 48 h later. We chose immune deficient mice as recipients to minimize the immunogenic effects of hCD8. Mice transplanted with *BCR-ABL*/hCD8-only control-cotransduced cells developed fatal myeloproliferative neoplasma (MPN) within 23 days following transplantation. The MPN was characterized by expansion of mature myeloid cells (Figure 5A far right) and their infiltration into BM, spleen, and liver (not shown) as observed in previous reports [18], [30]. Immunophenotypic analysis of BM cells showed a predominance of cells with high expression levels of Gr-1 and Mac-1, a feature of matured myeloid cells. The Gr-1/Mac-1 profile was comparable between hCD8-positive and -negative fractions, as expected for expression of hCD8 having no effect on cells (n = 2) (Figure 5A and Figure S6). Likewise, *AML1R80C* did not appreciably affect the matured myeloid-dominant phenotype (n = 3) (Fig. 5B and Figure S7), which was maintained following secondary transplantation (Figure 5B). In contrast, mice transplanted with *BCR-ABL*/*AML1K83Q-* or *BCR-ABL/*wt.*AML1*-cotransduced cells exhibited a marked difference between the hCD8-positive and -negative fraction with respect to immunophenotype. In cases involving *BCR-ABL/AML1K83Q*, the GFP+hCD8+ fraction was primarily composed of Lin^-/low^ cells, while the GFP+hCD8− fraction was composed of myeloid, B and erythroid/megakaryocytic cells (n = 3) (Figure 5C and Figure S8). The GFP+hCD8+ fraction in recipients of *BCR-ABL/*wt.*AML1-*transduced cells was primarily composed of Lin^-/low^ and/or CD41+ cells (Figure 5D and Figure S9), while the GFP+hCD8− fraction was primarily composed of myeloid and erythroid/megakaryocytic cells (n = 3) (Figure 5D and Figure S9). These findings suggest that expression of wt. AML1 or AML1K83Q led to the emergence of cells with a distinct phenotype among *BCR-ABL*+ cells. We then tested whether the *BCR-ABL+AML1K83Q+* or *BCR-ABL+*wt.*AML1+* cells exhibited a growth advantage over *BCR-ABL*-only-transduced cells. To this end, secondary transplantation experiments were performed. In the secondary recipient mice, *BCR-ABL/AML1K83Q* -cotransduced cells showed a relative expansion over *BCR-ABL*-only cells in 3 of 3 cases (Figure 5C and Figure S8) compared with primary recipients, but only one case showed such an expansion when compared with cells used for primary transplantation. This was also the case for *BCR-ABL*/wt.*AML1*; one of 3 cases showed expansion when compared with cells used for primary transplantation (Figure 5D and Figure S9). These findings suggest that wt.AML1 and AML1K83Q, but not AML1R80C, have the capacity, at least partly, to contribute to the manifestation of a BC-like phenotype (emergence of cells of a distinct phenotype) (Figure S10 summarizes compositions of cells in primary and secondary recipients). However, they are insufficient to induce a fully transformed BC-like disease, which probably reflects the need for additional genetic and/or epigenetic changes. These caveats aside, it is noteworthy that one of 3 cases involving of *BCR-ABL/AML1K83Q-*co-transduced cells and one of 3 cases involving *BCR-ABL/*wt.*AML1-*co-transduced cells showed a marked predominance of GFP+hCD8+ cells in secondary recipients (Figure 5C, D) compared with cells used for primary transplantation. Mice showed hepatosplenomegaly and mild lung hemorrhage (Figure S11A), and histological examination revealed infiltration of leukemic cells into organs such as spleen and liver (Figure S11B). Flow cytometric analysis of the spleen cells of diseased mice showed that the majority of cells from *BCR-ABL/AML1K83Q* mice were B220+/c-kit+ immature B cells (Figure 5C), and those from *BCR-ABL/*wt.*AML1* mice were CD41-positive cells (Figure 5D). These results suggest that *BCR-ABL/AML1K83Q* and *BCR-ABL/*wt.*AML1* respectively induced lymphoblastic and megakaryoblastic leukemia, and the morphology of cells was compatible with these phenotypes. Although reasons for the emergence of c-kit+ B-cell leukemia, the phenotype of which was not obvious in the primary recipient (Figure 5C), are not clear from the present study, the overall findings suggest the potential of *AML1K83Q* and wt.*AML1* to contribute to a BC-like disease.

## Discussion

Molecular mechanisms underlying the disease progression of CML are largely unknown, but comparison of BC with CP following deep sequencing and gene expression analysis may provide clues. Several reports have revealed the presence of recurrent somatic mutations specific to BC [12], [16]–[18], [46] and elevated expression of a subset of genes in BC compared with CP [34], [47], [48]. In the study presented here, we searched for *AML1* mutations and quantitated *AML1* transcripts in BC samples. We found *AML1* mutations in three of 12 evaluable BC cases (25%) and elevated levels of transcripts of wt.*AML1* in BC when compared with CP and normal bone marrow samples. The latter finding is consistent with the results of our analysis of published data of a larger number of cases [40] (GSE1470). These findings suggest the association of mutated *AML1* and/or elevated expression of *AML1* with the disease progression of CML.

To explore the effect of wt. or mutant AML1s on disease progression, we used mouse hematopoietic cells to retrovirally express BCR-ABL and AML1s. Our results showed that among AML1 mutants tested, AML1K83Q and AML1R139G conferred a growth advantage upon BCR-ABL-expressing cells over *BCR-ABL*-alone control cells in culture. However, AML1R139G was much less potent regarding colony forming ability than AML1K83Q, suggesting a lower clonogenic potential. In contrast, AML1R80C and AML1D171N did not confer a growth advantage. Rather unexpectedly, wt.AML1 behaved similarly to AML1K83Q. Although AML1G190R and AML1A297LfsX7 mutants found in our clinical samples co-operated with BCR-ABL to confer growth advantage upon cells in culture (Figure S12), we focused on AML1K83Q, AML1R80C and AML1R139G mutants for detailed analysis because these mutations recurrently occur in BC (Table S2). Cells co-transduced with *AML1K83Q* or wt.*AML1* along with *BCR-ABL* were lethal following intravenous transfer into mice. *AML1R139G/BCR-ABL*-cotransduced cells were again less potent in regard to lethality. Furthermore, AML1K83Q and wt.AML1 in a BMT assay contributed to the induction of a blast-like phenotype in the presence of BCR-ABL expression, whereas AML1R80C did not produce such a response. The positivity of CD41 in the blast-like cells is consistent with the phenotype of leukemia that developed in BXH2 mice that overexpress AML1 [49]. These findings suggest that some, but not all, AML1 mutants have the capacity to contribute to a BC-like phenotype. Overexpression of wt.AML1 also contributes to the process. Our *in vitro* assay showed that AML1K83Q retained DNA binding activity and exhibited dose-dependent transactivation activity, albeit to a lesser extent compared with wt.AML1. On the other hand, AML1R80C and AML1D171N inhibited, and AML1R139G slightly stimulated, the activity of wt.AML1. Therefore, at first glance there seems to be a correlation between transactivation activity and the potential to contribute to a BC-like phenotype, but this interpretation might be too simplistic. AML1 is essential for the development of hematopoiesis, and altered AML1s are associated with many hematological malignancies [7], [8]. In a mouse BMT model, AML1D171N and AML1S291fsX300 induced lethal MDS/AML in mice within a year [13]. On the other hand, only 1 of 3 mice transplanted with cells transduced with *AML1A224fsX228*, which is closely related to *AML1S291fsX300* in structure, died during the same time period [50]. The AML1a isoform encompassing the N-terminal region comprising 250 amino acids is able to expand hematopoietic stem/progenitor cells, but does not cause fetal disease in mice [51]. Although AML1D171N, AML1S291fsX300, AML1A224fsX228 and AML1a all function against wt.AML1 in a dominant-negative fashion in an *in vitro* transactivation assay [15], [19], [52], the difference in phenotype manifested *in vivo* is large. Although the reasons for the difference are unknown, a subtle difference in the structure of the AML1 moiety may affect the phenotype. Difficulty in assigning wt.AML1 as a pro- or anti-oncogenic gene provides an additional layer of complexity [53]. For instance, while expression of wt.AML1 in human foreskin fibroblasts or mouse embryonic fibroblasts (MEFs) induces senescence, it promotes outgrowth of p53-null MEF [54], [55]. Although analysis of TLX1/TLX3-induced T-cell acute lymphoblastic leukemia suggests AML1 is an oncosuppressor [56], overexpression of wt.AML1 promotes the development of mouse T-cell lymphoma [57] and accelerates leukemogenesis in BXH2 mice [49]. While enforced expression of wt.AML1 promotes differentiation of mouse hematopoietic cells and compromises engraftment of hematopoietic cells *in vivo* following transplantation [51], our study shows that wt.AML1 confers upon cells a growth advantage in culture amid BCR-ABL expression, and induces a BC-like phenotype in mice. The overall findings suggest the context-dependency of AML1 function, and highlight the difficulty in deciphering the association between *in vivo* phenotypes and the results of *in vitro* assays.

A recent report by Zhao [18] shows that AML1 H78Q and AML1V91fsX94 have the capacity to induce BC-like disease in mice in collaboration with BCR-ABL. The two mutants dominant-negatively inhibit activity of wt.AML1 in an *in vitro* transactivation assay [18]. Our experiments show that while AML1K83Q retains transactivation activity and contributes to a BC-like phenotype in mice, AML1R80C dominant-negatively inhibits activity of wt.AML1 but does not contribute to a BC-like phenotype in mice. Zhao’s and our studies used the same reporter and the same cells for the *in vitro* transactivation assay. These findings indicate that AML1 mutants showing dominant-negative activity against wt.AML1 do not necessarily contribute to a BC-like phenotype. Given that a subtle structural difference in the AML1 moiety could lead to a difference in the penetrance of disease and phenotypes of modeled MDS in mice (see description above), it is important to investigate AML1 mutants individually for their capacity amid BCR-ABL expression to induce BC-like disease in mouse models. In addition, a difference in cooperative genes may impose additional factors for consideration [13]. The dominance of cells harboring the AML1R80C mutant in a clinical sample, despite the inability of the mutant to induce a BC-phenotype in a mouse model, also implies the requirement of an additional genetic alteration for the disease progression of CML. The low penetrance of the BC-like phenotype conferred by AML1K83Q and wt.AML1 upon secondary transplantation in our study is consistent with this notion. Indeed, Grossmann et al. [16] showed that all cases but one that harbor the *AML1* mutation displayed karyotypic abnormalities and/or mutations in *ASXL1, IKZF1, WT, TET2, IHD1* or *CBL* genes, thus pointing to the presence of additional genetic and epigenetic abnormalities. Here, the search for mutations focused on 11 genes (*RUNX1, IKZF, CBL, NRAS, KRAS, IDH1, IDH2, NPM1, TET2, WT1* and *p53*), and therefore leaves open the possibility that more genes are mutated.

Our examination of clinical samples revealed elevated levels of wt.*AML1* transcripts in BC compared with CP, and the presence of outlier patients in BC regarding *AML1* transcript levels. Elevated expression of wt.AML1 is known to confer upon BCR-ABL-expressing cells resistance to imatinib [27]. We showed that shRNA-mediated silencing of AML1 attenuated growth of CML-BC cell lines. Although our preliminary experiments employing three BC cell lines suggest that moderate knock-down of AML1 expression is not sufficient to render the cells more sensitive to imatinib (Figure S13), the overall findings may imply that elevated expression of wt.AML1 could in part lead to imatinib-resistance, and culminates in disease progression to BC. Although the mechanisms underlying increased expression of AML1 are currently unknown, monitoring the amounts of AML1, as well as the emergence of its mutants along with additional genetic changes, may provide a better understanding of the roles of altered AML1 in the disease progression of CML.

## Supporting Information

Figure S1 *AML1* mRNA levels of CML patient samples. Data published by Radich et al. [40] were obtained (ONCOMINE) (https://www.oncomine.org/resource/login.html) and analyzed for statistical differences. The bottom side of the box represents the first quartile, and the third quartile is represented by the top side. The vertical width of the box represents the inter-quartile deviation. The horizontal line inside the box is the median. The vertical lines protruding from the box extend to the minimum and maximum values of the data set. The values plotted above the top whiskers are outliers. The difference in mRNA levels among CP, AP and BC cases were statistically significant (p = 0.00014) (Kruskal-Wallis rank sum test). Pairwise comparisons were made using the Mann-Whitney U-test and *post hoc* analysis with P-value adjustment using Holm’s method.
(TIF)

Figure S2 Western blot analysis of BC cell lines infected with shRNA viruses for AML1. MegA2, Nalm1 and MegO1 cells were infected with the indicated shRNA for AML1(shRNA1 and shRNA2) and luciferase (control), and subjected to Western analysis with anti-AML1 antibody. Anti-tubulin blot was included to allow comparison of amounts of loaded protein. Relative expression levels of AML1 are also presented.
(TIF)

Figure S3 Sustained growth of cells cotransduced with *BCR-ABL* (B/A) and either *NUP98-HOXA9*, *Bmi1* or *Hes1* in the liquid culture without cytokine supplementation. Cells were counted every other day with the use of trypan blue.
(TIF)

Figure S4 Cytokine-free liquid culture of mouse hematopoietic progenitor cells transduced with wt.AML1 and AML1 mutants. Lin-negative, Sca-1-negative progenitor cells were transduced with the indicated AML1, selected for puromycin-resistance and cultured without cytokines. Note that cell numbers rapidly decreased due to cell death.
(TIF)

Figure S5 Kaplan-Meier survival curves of recipient mice of progenitor cells that were transduced with the indicated retroviral constructs and grown in cytokine-free culture.
(TIF)

Figure S6 Flow cytometric analysis of bone marrow (BM) cells from a mouse receiving BM cells that were transduced with *BCR-ABL* and hCD8-only control.
(TIF)

Figure S7 Flow cytometric analysis of spleen (SP) cells from 2 mice transplanted with BM cells that were transduced with *BCR-ABL* and *AML1R80C*.
(TIF)

Figure S8 Flow cytometric analysis of spleen (SP) cells from 2 mice transplanted with BM cells that were transduced with *BCR-ABL* and *AML1K83Q*.
(TIF)

Figure S9 Flow cytometric analysis of spleen (SP) cells from 2 mice transplanted with BM cells that were transduced with *BCR-ABL* and wt.*AML1*.
(TIF)

Figure S10 Compositions of cell fractions transduced with BCR-ABL (GFP) and/or AML1 (hCD8). Indicated fractions in spleen cells were analyzed for cell compositions (Gr1+/Mac1+, Ter119+, CD41+, B220+, CD3+, and others) following primary and secondary transplantations. * indicates fractions with insufficient number of cells to evaluate.
(TIF)

Figure S11 (A) Photographs showing hepatosplenomegaly of representative secondary recipient mice transplanted with *BCR-ABL*/*AML1K83Q*- and *BCR-ABL*/wt.*AML1*-transduced cells.
(TIF)

Figure S12 Analysis of functions of AML1R135EfsX42, AML1A297LfsX7 and AML1G190R. (A) Transactivation potentials of the indicated AML1 mutants. Assay was performed as described in the legend to Figure 3C, and relative luciferase activities are presented. (B) Cytokine-independent growth of progenitor cells transduced with BCR-ABL and the indicated AML1 mutant. Assay was performed as described in the legend to Figure 4A. (C) Western analysis of cells used in (B). Anti-Flag blot detected expected sizes of AML1 mutants. (D) Flow cytometric analysis of cells in (B). Cells co-transduced with BCR-ABL and AML1G190R or AML1A297LfsX7 that gained a growth advantage over control were analyzed for expressions of the indicated molecules.
(TIF)

Figure S13 Analysis of sensitivity of cells to imatinib. (A)(B) MegA2, MegO1 and Nalm1 cells were infected with shRNA virus for AML1 (AML1–1 and AML1–2 used in Figure 2) and luciferase (control), and treated with indicated concentrations of imatinib for 3 days. (A) GFP co-expressed with shRNA allowed monitoring percentage of surviving cells. (B) Note that shRNA for AML1 had no appreciable effects on sensitivity of the cells to imatinib treatment.
(TIF)

Table S1 Summary of BC samples used for quantification of AML1 transcript in Figure 1.
(TIF)

Table S2 Reported mutations of *AML1* in CML-BC patients.
(TIF)
