# Supplementary figures and images for: Functionally Deregulated AML1/RUNX1 Cooperates with BCR-ABL to Induce a Blastic Phase-Like Phenotype of Chronic Myelogenous Leukemia in Mice
Source: PLoS One. 2013 Sep 30;8(9):e74864. doi: 10.1371/journal.pone.0074864 (PMC3787010; doi:10.1371/journal.pone.0074864)

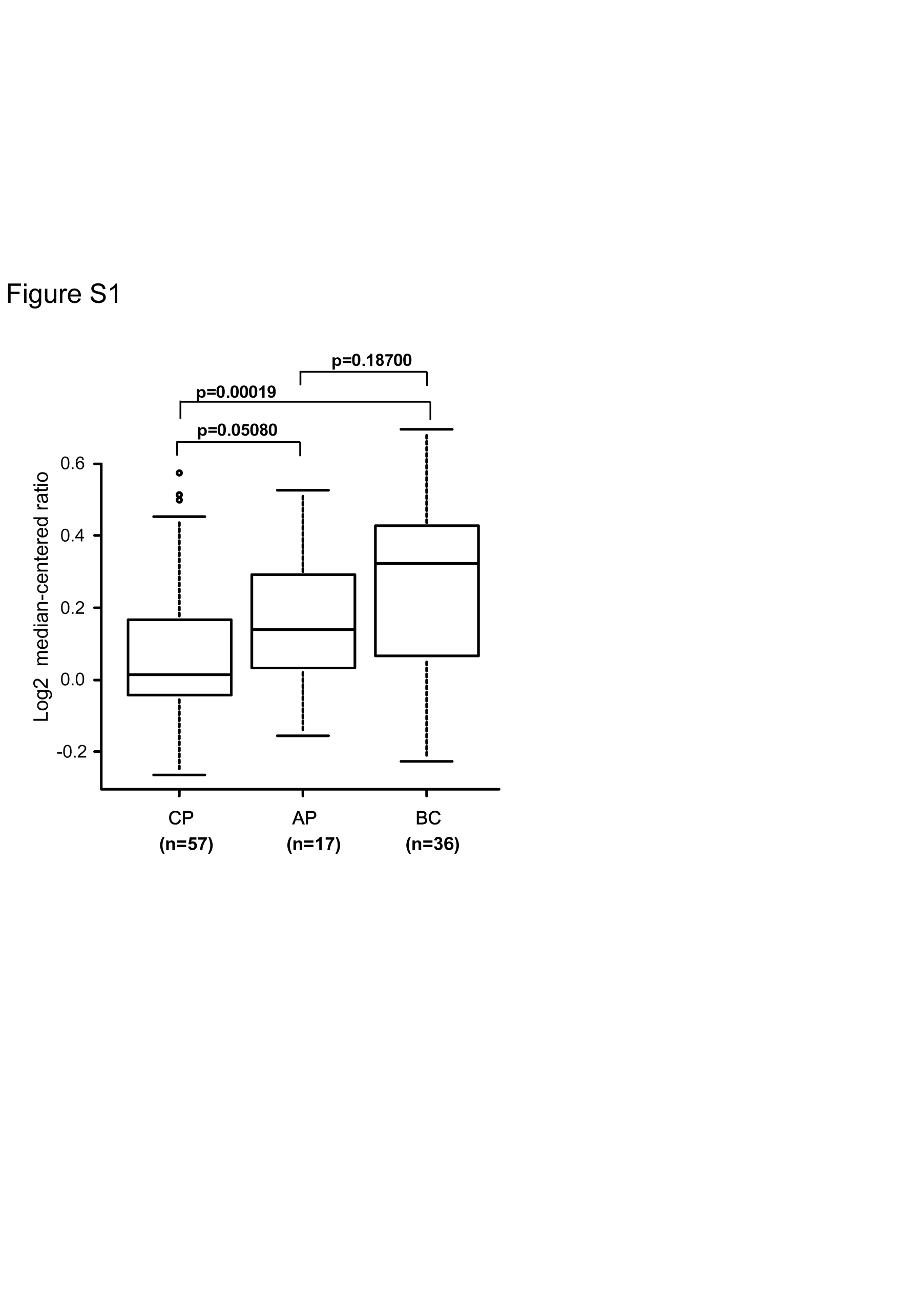

Supplement: Figure S1 — AML1 mRNA levels of CML patient samples. Data published by Radich et al. [40] were obtained (ONCOMINE) (https://www.oncomine.org/resource/login.html) and analyzed for statistical differences. The bottom side of the box represents the first quartile, and the third quartile is represented by the top side. The vertical width of the box represents the inter-quartile deviation. The horizontal line inside the box is the median. The vertical lines protruding from the box extend to the minimum and maximum values of the data set. The values plotted above the top whiskers are outliers. The difference in mRNA levels among CP, AP and BC cases were statistically significant (p = 0.00014) (Kruskal-Wallis rank sum test). Pairwise comparisons were made using the Mann-Whitney U-test and post hoc analysis with P-value adjustment using Holm’s method. (TIF) [file pone.0074864.s001.tif]

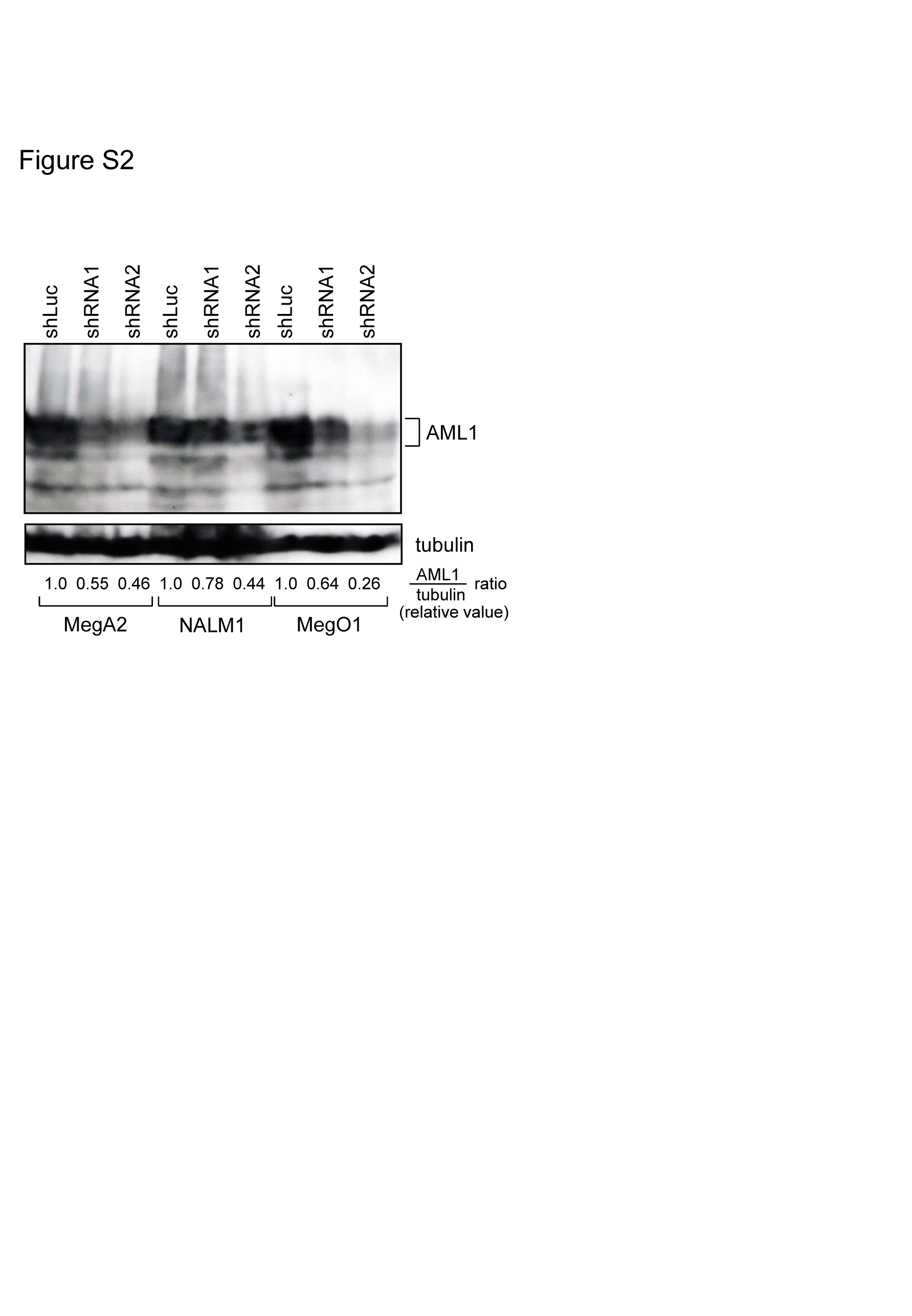

Supplement: Figure S2 — Western blot analysis of BC cell lines infected with shRNA viruses for AML1. MegA2, Nalm1 and MegO1 cells were infected with the indicated shRNA for AML1(shRNA1 and shRNA2) and luciferase (control), and subjected to Western analysis with anti-AML1 antibody. Anti-tubulin blot was included to allow comparison of amounts of loaded protein. Relative expression levels of AML1 are also presented. (TIF) [file pone.0074864.s002.tif]

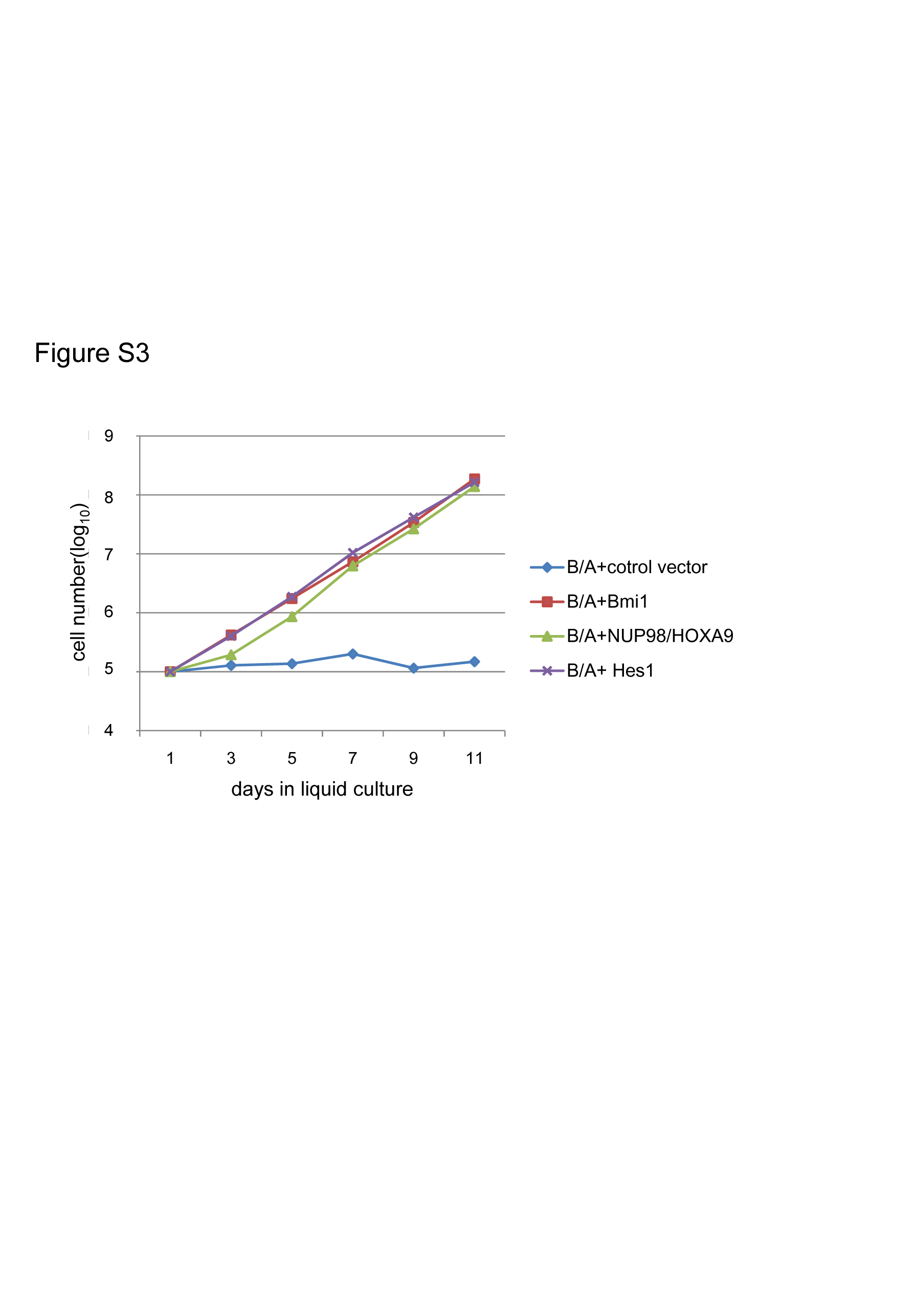

Supplement: Figure S3 — Sustained growth of cells cotransduced with BCR-ABL (B/A) and either NUP98-HOXA9, Bmi1 or Hes1 in the liquid culture without cytokine supplementation. Cells were counted every other day with the use of trypan blue. (TIF) [file pone.0074864.s003.tif]

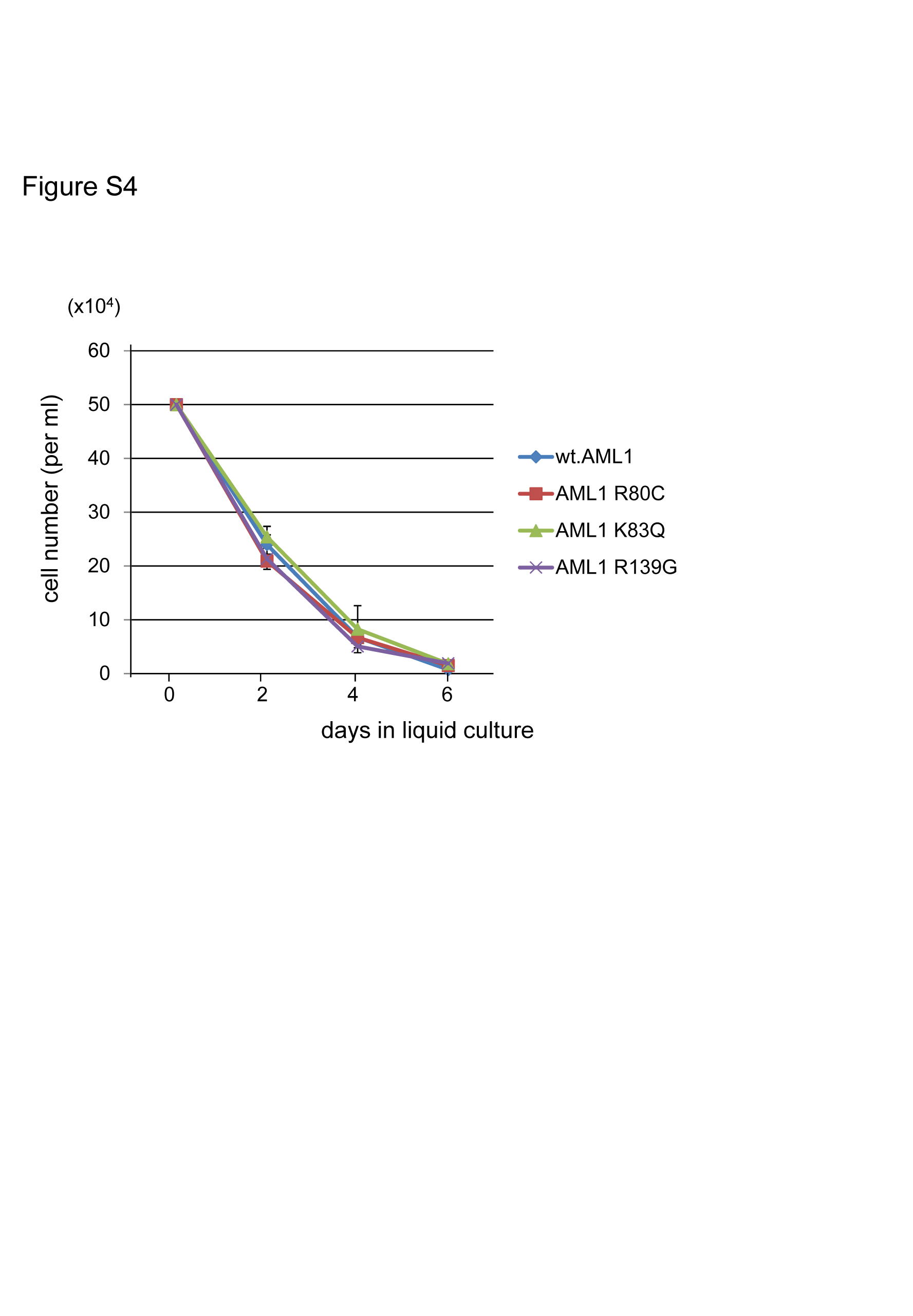

Supplement: Figure S4 — Cytokine-free liquid culture of mouse hematopoietic progenitor cells transduced with wt.AML1 and AML1 mutants. Lin-negative, Sca-1-negative progenitor cells were transduced with the indicated AML1, selected for puromycin-resistance and cultured without cytokines. Note that cell numbers rapidly decreased due to cell death. (TIF) [file pone.0074864.s004.tif]

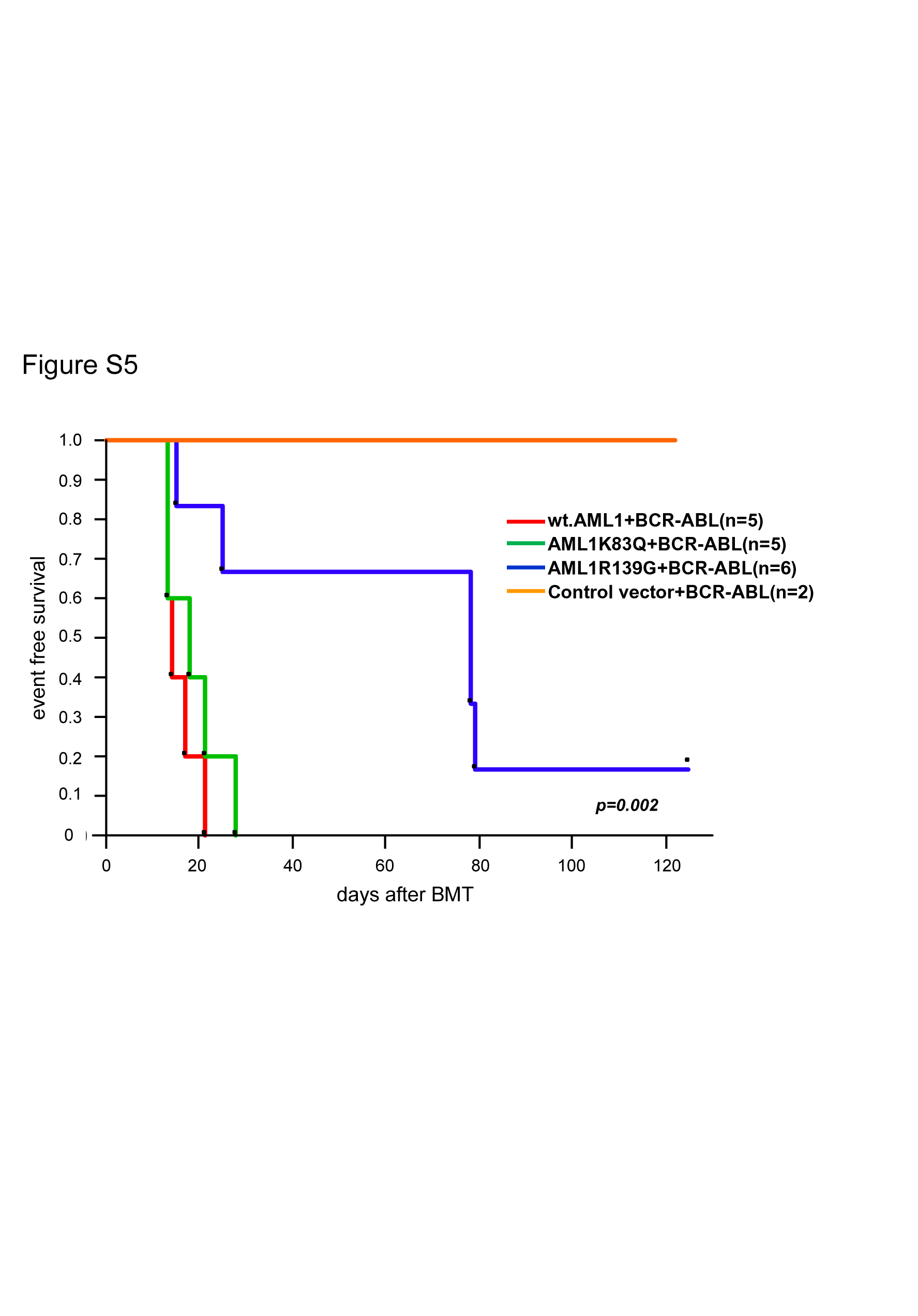

Supplement: Figure S5 — Kaplan-Meier survival curves of recipient mice of progenitor cells that were transduced with the indicated retroviral constructs and grown in cytokine-free culture. (TIF) [file pone.0074864.s005.tif]

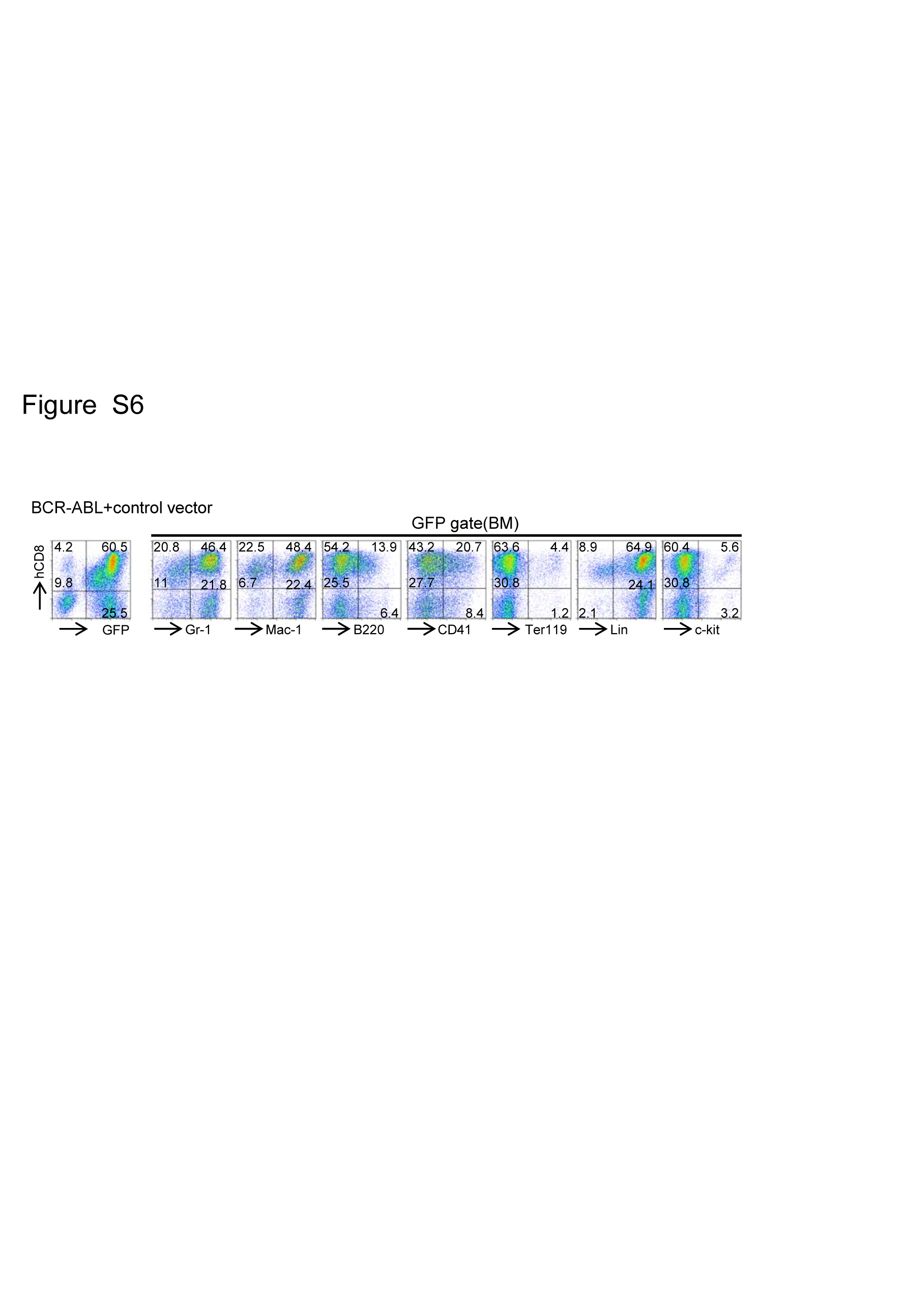

Supplement: Figure S6 — Flow cytometric analysis of bone marrow (BM) cells from a mouse receiving BM cells that were transduced with BCR-ABL and hCD8-only control. (TIF) [file pone.0074864.s006.tif]

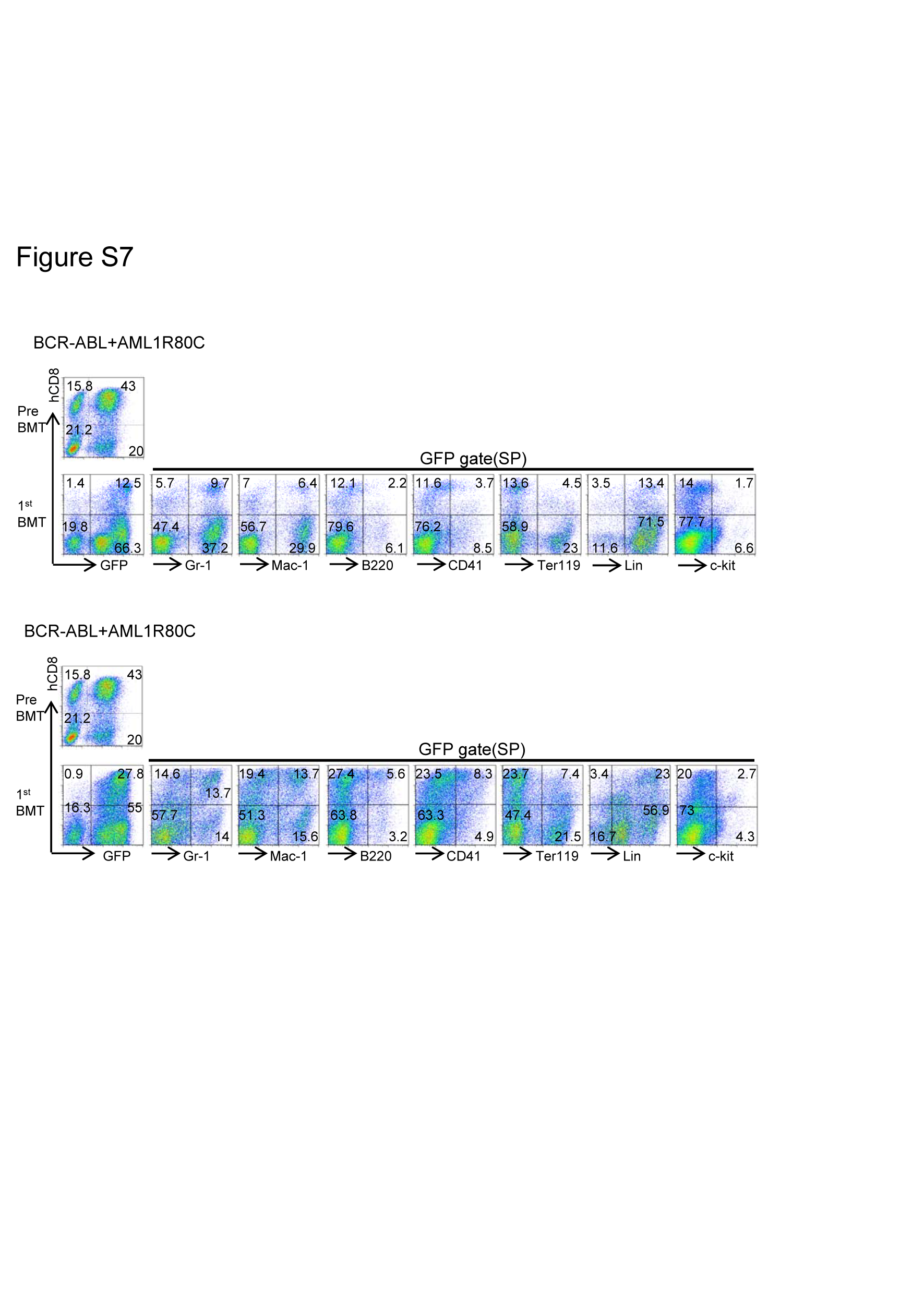

Supplement: Figure S7 — Flow cytometric analysis of spleen (SP) cells from 2 mice transplanted with BM cells that were transduced with BCR-ABL and AML1R80C. (TIF) [file pone.0074864.s007.tif]

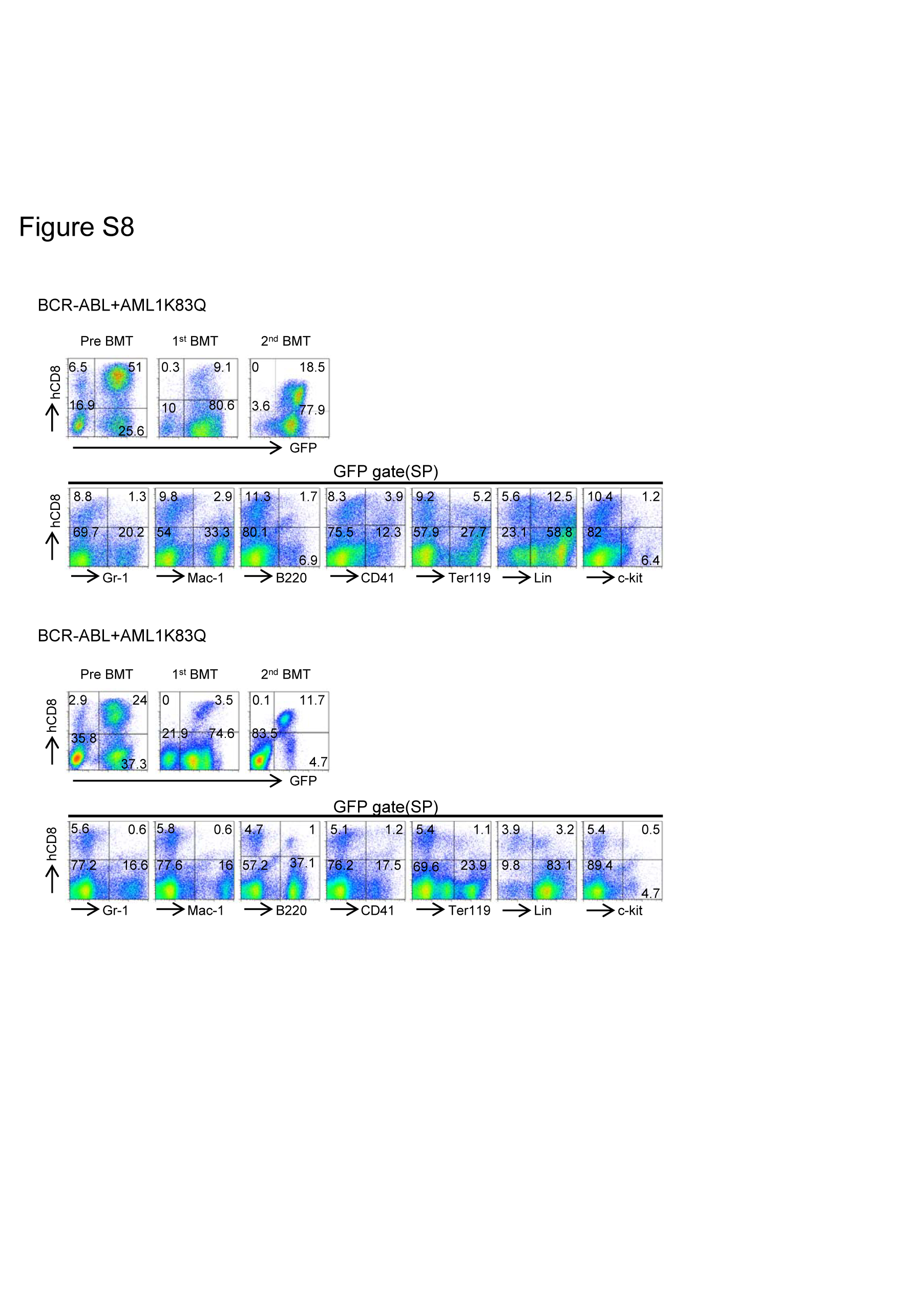

Supplement: Figure S8 — Flow cytometric analysis of spleen (SP) cells from 2 mice transplanted with BM cells that were transduced with BCR-ABL and AML1K83Q. (TIF) [file pone.0074864.s008.tif]

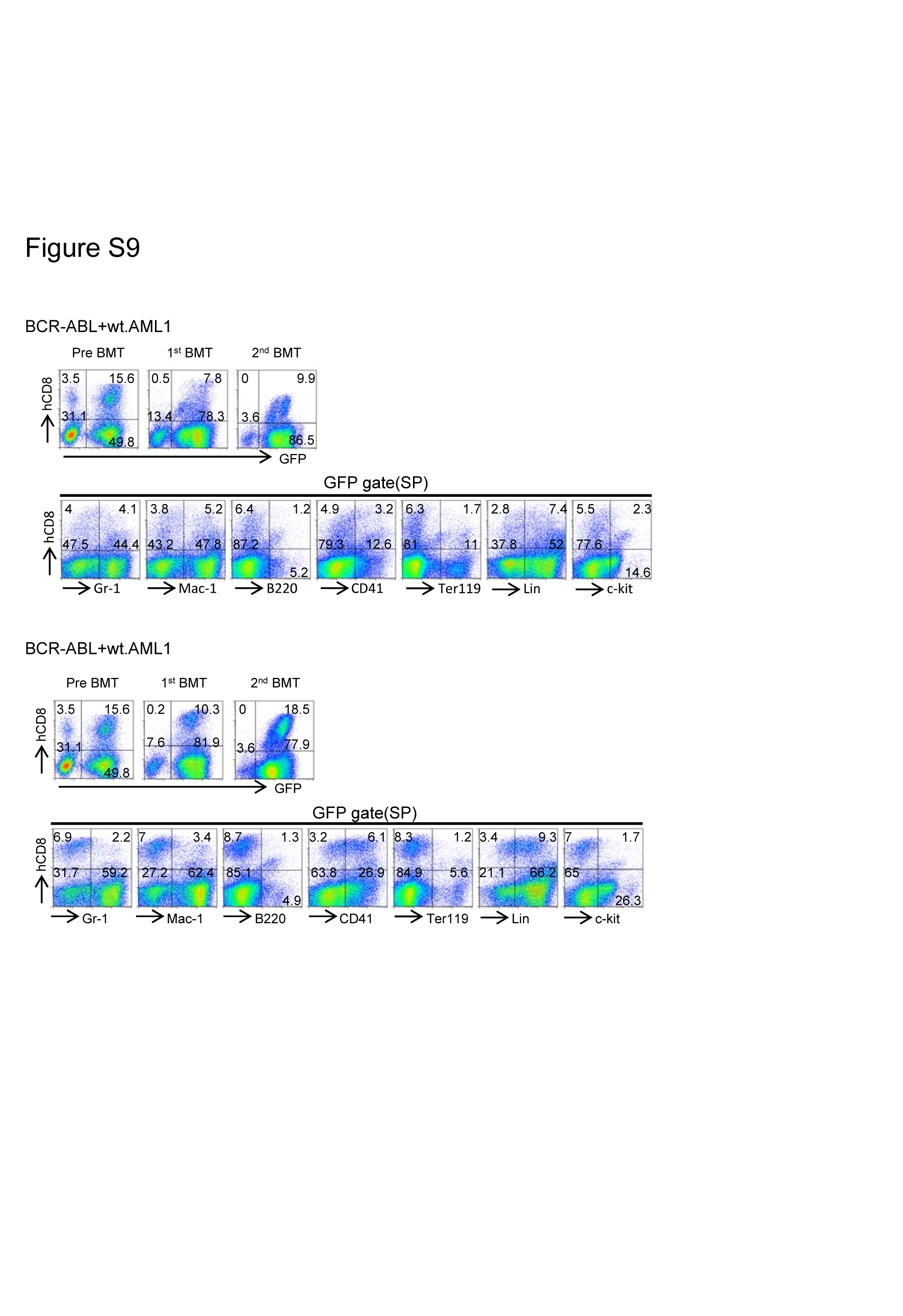

Supplement: Figure S9 — Flow cytometric analysis of spleen (SP) cells from 2 mice transplanted with BM cells that were transduced with BCR-ABL and wt.AML1. (TIF) [file pone.0074864.s009.tif]

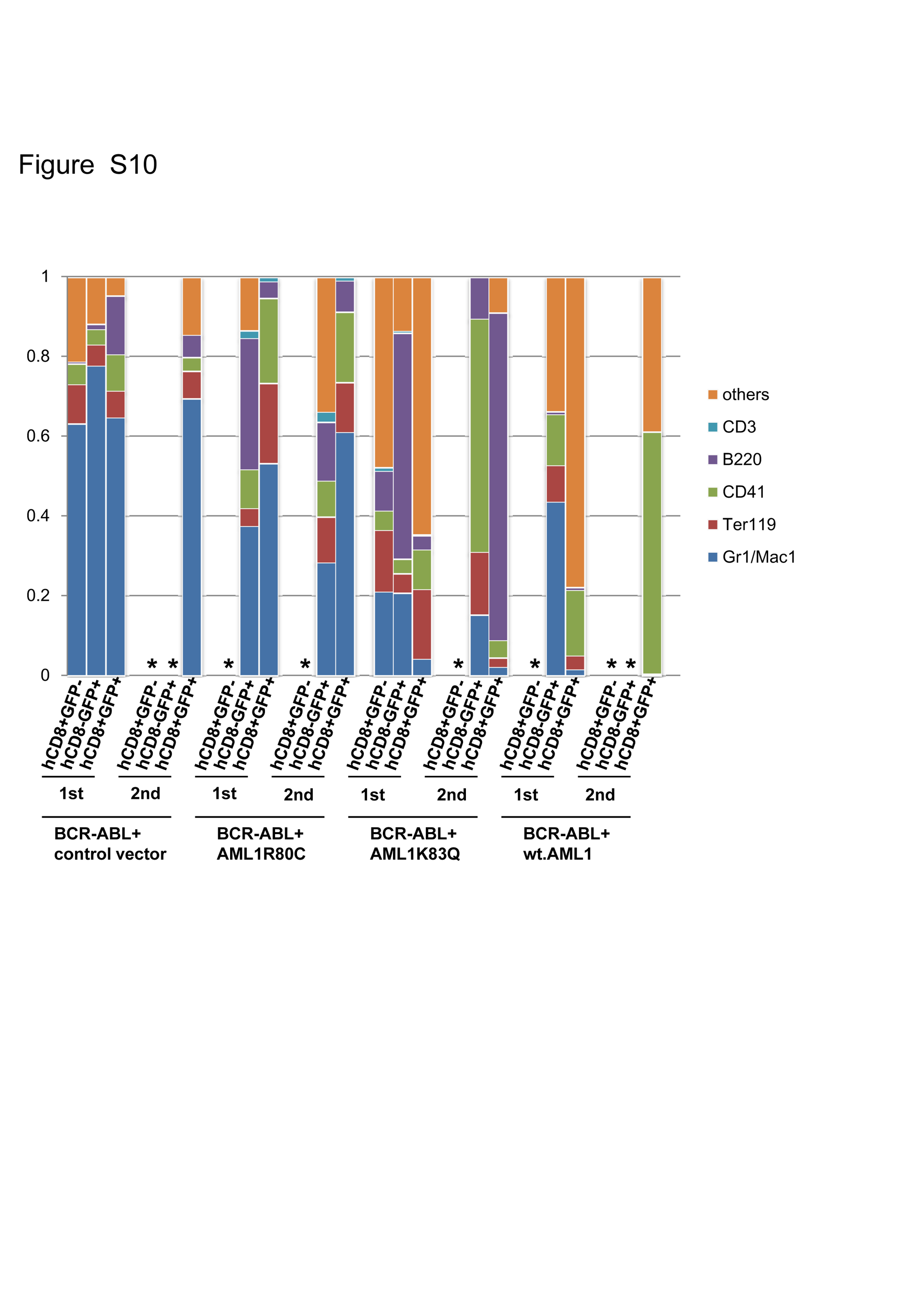

Supplement: Figure S10 — Compositions of cell fractions transduced with BCR-ABL (GFP) and/or AML1 (hCD8). Indicated fractions in spleen cells were analyzed for cell compositions (Gr1+/Mac1+, Ter119+, CD41+, B220+, CD3+, and others) following primary and secondary transplantations. * indicates fractions with insufficient number of cells to evaluate. (TIF) [file pone.0074864.s010.tif]

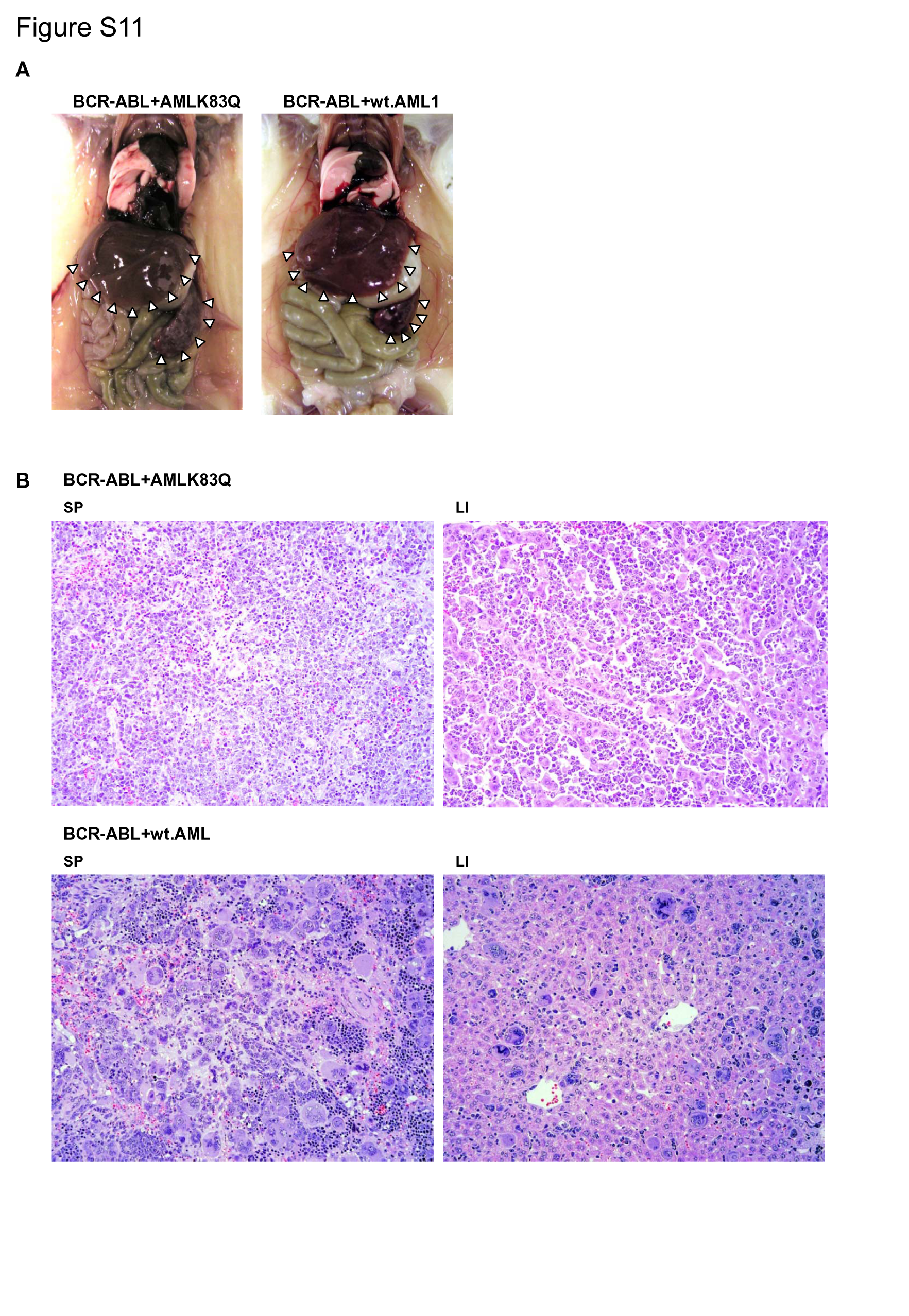

Supplement: Figure S11 — (A) Photographs showing hepatosplenomegaly of representative secondary recipient mice transplanted with BCR-ABL/AML1K83Q- and BCR-ABL/wt.AML1-transduced cells. (TIF) [file pone.0074864.s011.tif]

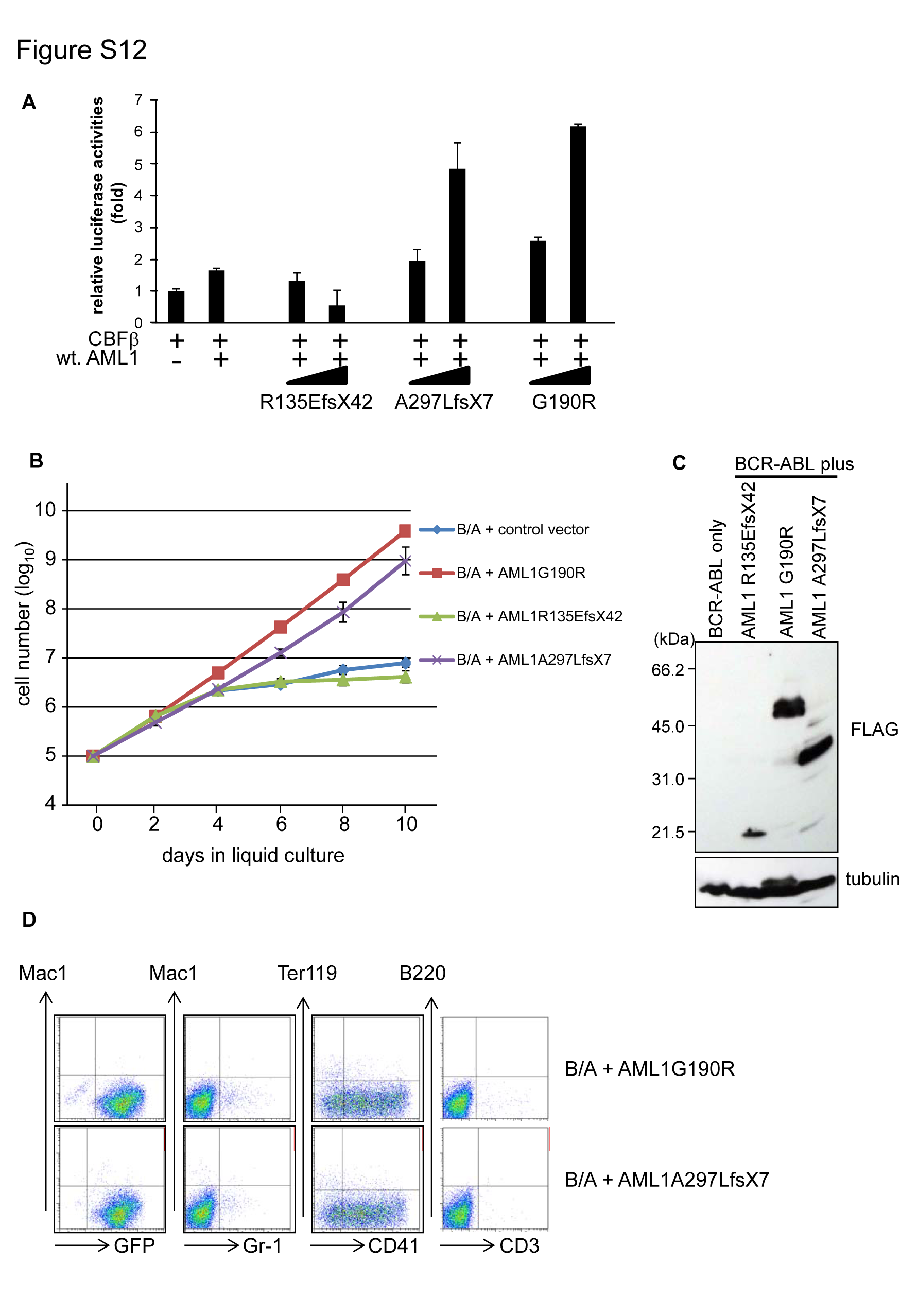

Supplement: Figure S12 — Analysis of functions of AML1R135EfsX42, AML1A297LfsX7 and AML1G190R. (A) Transactivation potentials of the indicated AML1 mutants. Assay was performed as described in the legend to Figure 3C, and relative luciferase activities are presented. (B) Cytokine-independent growth of progenitor cells transduced with BCR-ABL and the indicated AML1 mutant. Assay was performed as described in the legend to Figure 4A. (C) Western analysis of cells used in (B). Anti-Flag blot detected expected sizes of AML1 mutants. (D) Flow cytometric analysis of cells in (B). Cells co-transduced with BCR-ABL and AML1G190R or AML1A297LfsX7 that gained a growth advantage over control were analyzed for expressions of the indicated molecules. (TIF) [file pone.0074864.s012.tif]

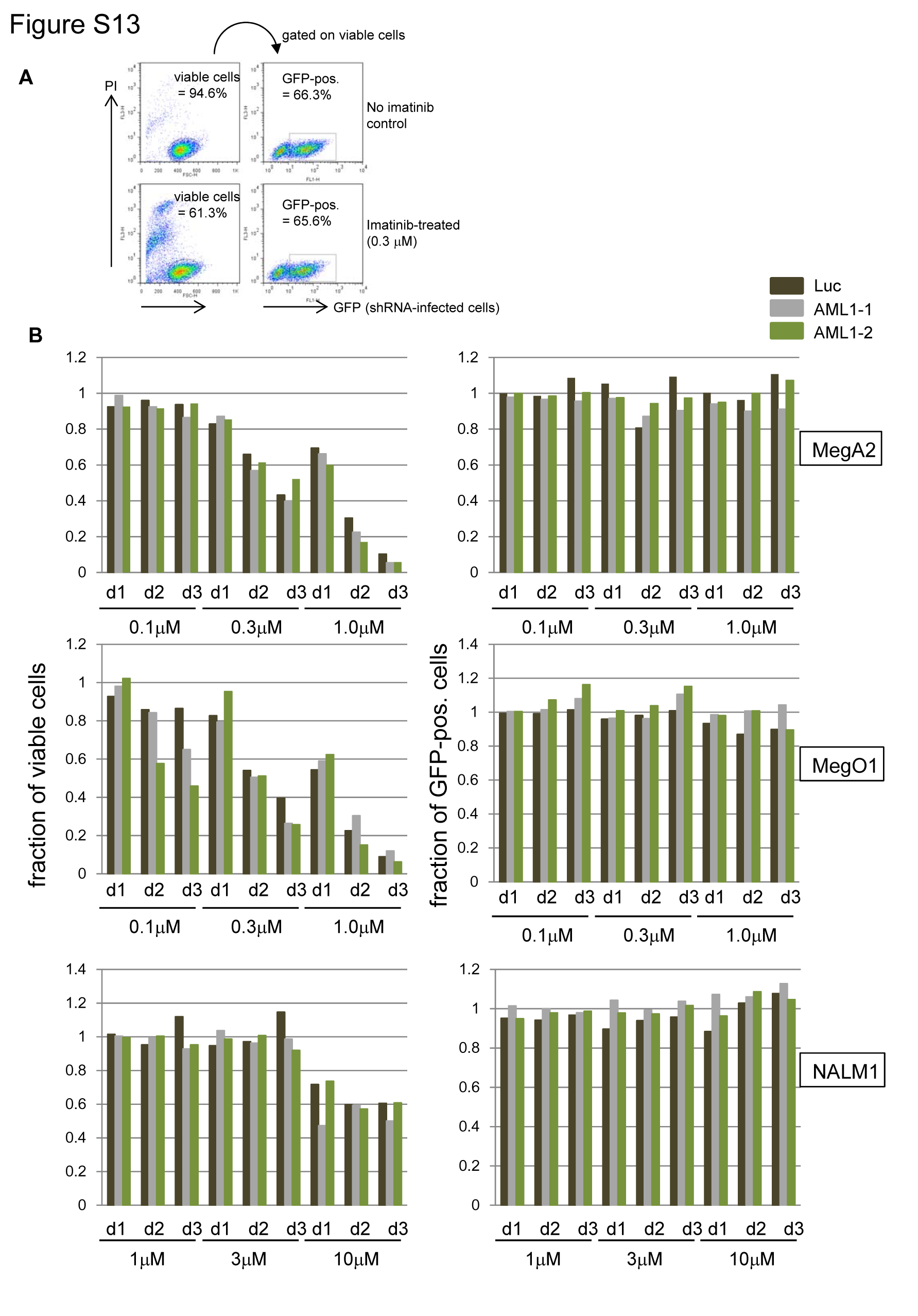

Supplement: Figure S13 — Analysis of sensitivity of cells to imatinib. (A)(B) MegA2, MegO1 and Nalm1 cells were infected with shRNA virus for AML1 (AML1–1 and AML1–2 used in Figure 2) and luciferase (control), and treated with indicated concentrations of imatinib for 3 days. (A) GFP co-expressed with shRNA allowed monitoring percentage of surviving cells. (B) Note that shRNA for AML1 had no appreciable effects on sensitivity of the cells to imatinib treatment. (TIF) [file pone.0074864.s013.tif]

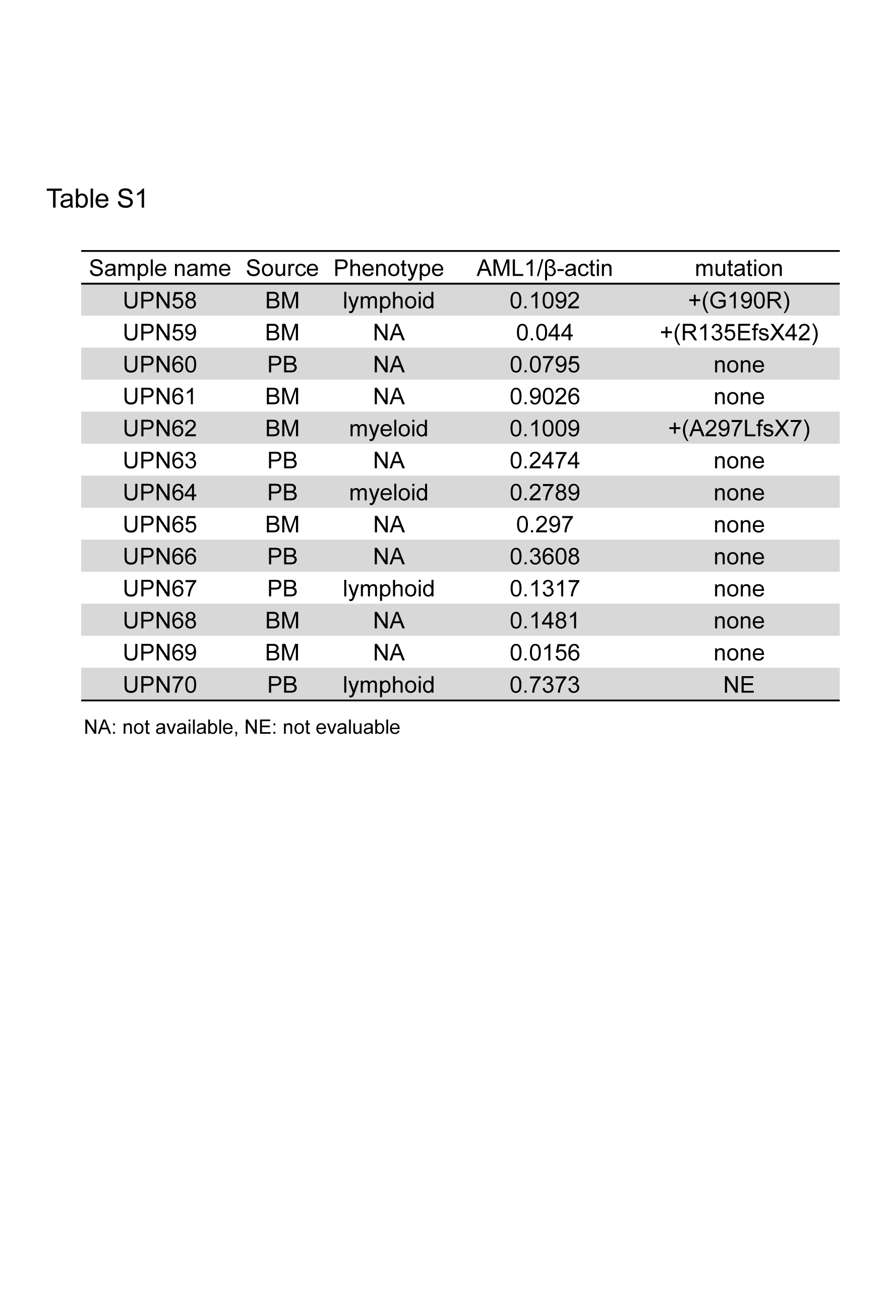

Supplement: Table S1 — Summary of BC samples used for quantification of AML1 transcript in Figure 1. (TIF) [file pone.0074864.s014.tif]

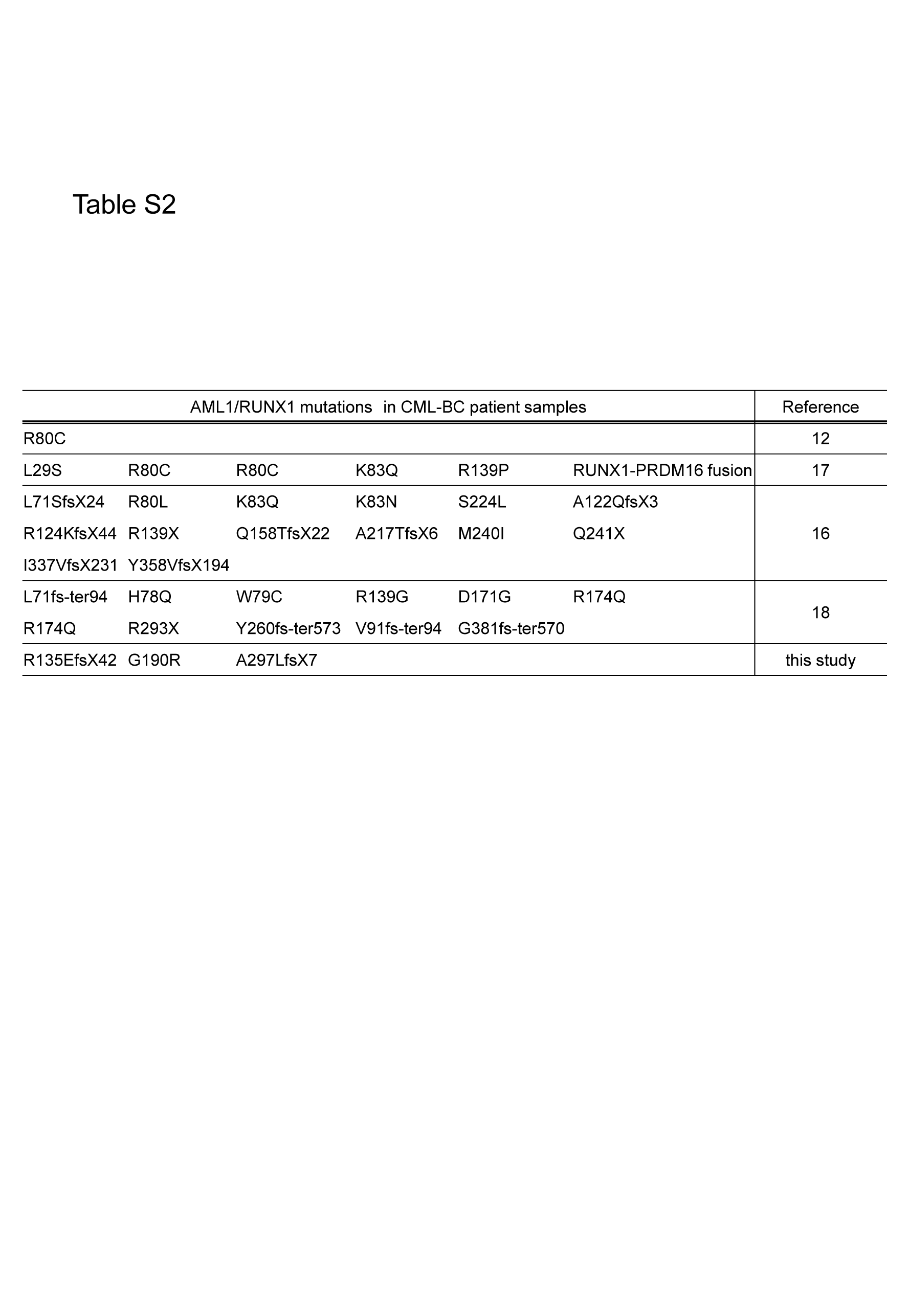

Supplement: Table S2 — Reported mutations of AML1 in CML-BC patients. (TIF) [file pone.0074864.s015.tif]
